# The Preservation of Muscle Mitochondrial Machinery During Hypometabolic Hibernation in Scandinavian Brown Bears ( *Ursus arctos* )

**DOI:** 10.1111/apha.70177

**Published:** 2026-02-23

**Authors:** Audrey Bergouignan, John Noone, Charlotte Brun, Laura Cussonneau, Alexandre Geffroy, Cecile Coudy‐Gandilhon, Isabelle Chery, Alina Lynn Evans, Jon Martin Arnemo, Jonas Kindberg, Guillemette Gauquelin‐Koch, Donal O'Gorman, Etienne Lefai, Fabrice Bertile

**Affiliations:** ^1^ Université de Strasbourg, CNRS, IPHC UMR 7178 Strasbourg France; ^2^ Division of Endocrinology, Metabolism and Diabetes Anschutz Health & Wellness Center, University of Colorado, Anschutz Medical Campus Aurora Colorado USA; ^3^ Department of Physical Education and Sport Sciences Faculty of Education and Health Sciences, University of Limerick Limerick Ireland; ^4^ Proteomics French Infrastructure, PROFI‐CORE UAR2048 Strasbourg France; ^5^ Université Clermont Auvergne, INRAE, Unité de Nutrition Humaine Clermont Ferrand France; ^6^ Department of Forestry and Wildlife Management Faculty of Applied Ecology and Agricultural Sciences, Inland Norway University of Applied Sciences Elverum Norway; ^7^ Norwegian Institute for Nature Research Trondheim Norway; ^8^ Department of Wildlife, Fish and Environmental Studies Swedish University of Agricultural Sciences Umeå Sweden; ^9^ Centre National d'Etudes Spatiales, CNES Paris France; ^10^ School of Health and Human Performance Dublin City University Dublin Ireland

**Keywords:** bear, electron transport chain, hibernation, mitochondria, muscle physiology, oroboros, proteomics

## Abstract

**Aim:**

Unlike humans, brown bears (*Ursus arctos*) uniquely preserve skeletal muscle mass and function during months of hibernation despite prolonged fasting and inactivity. We investigated how mitochondrial energetics respond in skeletal muscle to support this remarkable resilience.

**Methods:**

Muscle biopsies from eight wild brown bears were collected during hibernation and again in the active summer season. We assessed mitochondrial respiration using high‐resolution respirometry and evaluated changes in protein expression, enzyme activity, and mitochondrial content through proteomics, Western blotting, enzymatic assays, and DNA quantification.

**Results:**

Hibernation was associated with lower mitochondrial respiratory capacity, largely due to a reduction in mitochondrial density rather than damage or dysfunction. Despite reduced SDH subunit expression in the whole skeletal muscle, SDH activity remained stable. This likely reflects post‐translational regulation and increased, or at least maintained, functional efficiency of the remaining Complex II, allowing mitochondrial respiration to shift toward Complex II‐mediated electron entry during hibernation. Proteomic analyses revealed targeted adjustments that maintained energy efficiency, supported both fat and carbohydrate oxidation at low temperatures, and minimized energy loss. Additionally, selective downregulation of mitochondrial dynamic proteins may help protect against muscle degradation.

**Conclusion:**

These findings highlight a temperature‐sensitive, multifaceted strategy that preserves mitochondrial energetics during prolonged inactivity, despite reduced mitochondrial density. The selective maintenance of electron flow and fuel flexibility offers novel insights for mitigating muscle wasting in sedentary or immobilized humans.

## 1 Introduction

Endothermy has evolved in various taxonomic groups, including insects, fish, reptiles, birds, and mammals, as well as in some plants. To maintain their body temperature at high, relatively constant levels regardless of external influence, endotherms are able to generate heat from metabolic activity, muscle contraction, and/or the uncoupling of oxidative phosphorylation. However, the high energy costs associated with these processes mean that food must be available at all times, especially when environmental conditions deteriorate, such as a sustained drop in ambient temperatures. In regions where ambient temperatures vary radically from season to season and where food is in short supply at the same time, a number of endotherms (thus considered heterothermic) are able to modify their physiology to enter more or less prolonged periods of hypometabolism, such as hibernation [1]. In short, a drastic and controlled reduction in metabolic rates associated with low body temperature is observed during periods of torpor, a state of suspended animation. In most hibernators, periods of torpor alternate with periodic arousals, during which body temperature returns to euthermia. In other species, torpor is maintained throughout the hibernation period, such as in the bear (*Ursidae* family). The main physiological characteristics of metabolic suppression during hibernation have been reviewed in detail elsewhere [2, 3, 4, 5]. Yet briefly, hibernation is associated with a marked reduction in energy expenditure. This occurs primarily through depression of the thermoregulatory set point and the resulting decrease in thermogenesis [4], which is further amplified by the Q_10_ effect of lowered body temperature. Additional contributions arise from reduced physical activity, decreased heart and respiratory rates, suppression of innate immunity, drastically reduced kidney function, and cellular mechanisms that limit mitochondrial ROS production [3].

Metabolic rate, body mass, and body temperature are intimately intertwined. According to previous reports, minimal metabolic rates in the torpor state drop to an average of 4% of basal rates (range 1% to 20%) for small hibernators weighing less than 5 kg (range 4 g to 3.4 kg) with an average body temperature of 4°C (range −2.9°C to 15°C) [6]. In large hibernators such as bears (in the 80–100 kg body mass range), minimum metabolic rates during torpor have been recorded at 19%–25% of basal rates, with body temperature regulated between 29°C and 36°C [6, 7]. These values highlight an important distinction between small and large hibernators. In small species, deep torpor is accompanied by a profound drop in body temperature and metabolic rate that follows expected allometric scaling relationships (metabolic rate × body mass^0.75^). In contrast, bears suppress metabolism far less than predicted for their body size, maintaining comparatively high body temperatures. Thus, their torpid metabolic rates do not follow the allometric patterns observed in smaller hibernators [3]. Both temperature‐dependent and temperature‐independent mechanisms seem to be at play during hibernation, as previously suggested for hibernators weighing less than 10 kg [8, 9]. In bears, metabolic suppression during hibernation has been attributed mainly to mechanisms independent of the slightly lowered body temperature [7]. The various temperature‐independent mechanisms that may be involved in the suppression of metabolism and energy savings during hibernation have recently been reviewed [3, 5]. As the major ATP‐producing factory in the cell, mitochondria, of course, play a central role in metabolic suppression during hibernation, as documented mainly in small hibernating rodents [4].

Although ATP yield per unit oxygen can vary depending on substrate type and coupling efficiency [10], mitochondrial respiration accounts for ~90% of total oxygen consumption [11], making oxygen consumption a useful proxy for ATP production. Suppression of liver mitochondrial respiration (by up to 70%) has been reported during the torpor phase in various small hibernating rodents, with reversal during the arousal phases, but to different degrees depending on the animal species and assay conditions [4]. In thirteen‐lined ground squirrels ( *Ictidomys tridecemlineatus* ), mitochondrial respiration is also reduced in skeletal and cardiac muscles, albeit to a more modest extent (−30%) during hibernation [12], but it remains unchanged in hibernating arctic ground squirrels ( *Spermophilus parryii* ) [13]. To our knowledge, only one study has assessed cellular respiration in bears—the grizzly bear ( *Ursus arctos horribilis* ), reporting lower oxygen consumption in adipocytes from hibernating animals compared to cells collected from summer‐active bears, reflecting reduced adipocyte mitochondrial activity at the cellular level (*n* = 2) [14]. In bear skeletal muscles, metabolic depression is supported by a drop in the expression of nearly all subunits of the mitochondrial respiratory chain complexes and of the proteins of the tricarboxylic acid cycle (TCA) during hibernation, while the upregulation of pyruvate dehydrogenase kinase isoenzyme 4 (PDK4) and concomitant downregulation of pyruvate dehydrogenases A1 and B (PDHA1 and B) suggests that carbohydrate use for fueling the TCA cycle is disrupted [15]. Yet, how skeletal muscle mitochondria behave during hibernation remains to be determined.

Similar to other hibernators [3], bears exhibit a remarkable physiological response during hibernation: despite undergoing prolonged periods of fasting and physical inactivity during hibernation, they do not experience significant skeletal muscle atrophy [16]. This natural resistance to muscle wasting makes hibernating brown bears ( *Ursus arctos* ) a compelling model for identifying novel therapeutic targets to prevent or treat muscle atrophy in humans—a condition affecting millions of people worldwide [17], and projected to become increasingly more prevalent. Muscle atrophy leads to reduced strength, mobility, and quality of life, and is associated with fatigue, frailty, metabolic disturbances, and slower recovery from illness [18]. One of the key drivers of muscle wasting is mitochondrial dysfunction [19], and improving mitochondrial oxidative capacity is considered a promising therapeutic avenue [20]. From previous works, we have already identified several mechanisms that likely contribute to muscle preservation in hibernating bears, these include reduced oxidative stress levels, induction of a myogenic miRNA program, modulation of TGFbeta/BMP balance in favor of BMP at the transcriptomic level and the existence of anti‐proteolytic compounds circulating in the serum of winter bears (for a review, see [3]).

In this current study, we have examined skeletal muscle mitochondrial respiration in hibernating bears using a combination of proteomic and functional assessments of isolated mitochondria and muscle fibers, enzymatic assays of succinate dehydrogenase (SDH), and quantification of mitochondrial abundance. During hibernation, we observed lower mitochondrial respiratory rates with both carbohydrate and lipid substrates, alongside a reduction in mitochondrial content, while the enzymatic machinery remained largely intact. Notably, the potential for maximal SDH activity was preserved, supporting mitochondrial energetics at low temperatures. It should be noted, however, that as such this measurement reflects potential rather than physiological flux. Furthermore, fatty acid oxidation remained active in winter muscle under cold exposure. These findings suggest that bears maintain skeletal muscle function through strategic changes in mitochondrial functionality, conserving energy during hibernation while retaining the capacity for rapid muscle recovery—insights that could inform the development of interventions for skeletal muscle atrophy in humans.

## 2 Materials and Methods

### 2.1 Animals and Sample Collection

Fieldwork was carried out in 2018 and 2019 in Dalarna and Gävleborg Counties, Sweden. Eight brown bears (*Ursus arctos; 5 females and 3 males*) aged 2 years (except 2 females aged 3 years) were included in the study. Captures were performed as previously described [21], first during hibernation (February) by darting the animals directly in their dens, and the same animals were recaptured when active in June by darting from a helicopter. The mean body mass of hibernating bears (43.2 ± 4.2 kg) compared with summer‐active bears (46.3 ± 3.6 kg) was not significantly different (paired *t*‐test, *p* = 0.19) with individual data presented in Table S1.

Immobilization followed well‐established procedures [22], using a combination of medetomidine‐ketamine‐tiletamine‐zolazepam to anesthetize hibernating bears, whereas summer‐active bears were anesthetized with a medetomidine‐tiletamine‐zolazepam mixture. Once anesthetized, each bear was placed on an insulating blanket to collect tissue samples. Biopsies of skeletal muscle (*Vastus lateralis*) were collected, a piece of which (~50 mg) was immediately frozen on dry ice, another piece (~150–200 mg) was placed in an ice‐cold mitochondrial buffer (Mannitol 225 mM, Sucrose 75 mM, Tris 10 mM, EDTA 0.1 mM, pH = 7.4), and a third piece (~10–20 mg) was immediately placed in ice‐cold BIOPS solution (10 mM Ca–EGTA buffer, 0.1 M free calcium, 20 mM imidazole, 20 mM taurine, 50 mM potassium 2‐[N‐morpholino]‐ethanesulfonic acid, 0.5 mM dithiothreitol, 6.56 mM MgCl_2_, 5.77 mM ATP, and 15 mM phosphocreatine [PCr], pH 7.1). Ice‐cold samples were transported to the field station (approximately 30–60 min drive) in a cooler maintained at 4°C–5°C.

The study was approved by the Swedish Ethical Committee on Animal Experiments, Uppsala, Sweden (applications Dnr C3/2016 and Dnr C18/2015), the Swedish Environmental Protection Agency (NV‐0741‐18), and the Swedish Board of Agriculture (Dnr 5.2.18‐3060/17). All procedures complied with Swedish laws and regulations.

### 2.2 Preparation of Permeabilized Muscle Fiber Bundles

Muscle fiber bundles were gently teased apart in a petri dish containing ice‐cold BIOPS solution with fine‐nosed forceps and a dissecting microscope. Approximately 2–5 mg fiber bundles were permeabilized with agitation in saponin (5 mg/mL) dissolved in BIOPS for 30 min at 4°C and then washed once for 10 min at 4°C with MiR05 respiration medium (0.5 mM EGTA, 3 mM MgCl_2_·6H_2_O, 60 mM K‐lactobionate, 20 mM taurine, 10 mM KH_2_PO_4_, 20 mM HEPES, 110 mM sucrose, and 1 g/L BSA, pH 7.1), all on an orbital shaker. The permeabilized muscle fiber bundles were blotted, weighed using a fine balance, and placed immediately in the high‐resolution respirometry (HRR) chambers of an Oxygraph 2K (Oroboros Inc., Innsbruck, Austria) for analysis.

### 2.3 Preparation of Isolated Mitochondria

Isolated mitochondria were prepared from 150 to 200 mg of bear muscle tissue. Homogenization in ice‐cold mitochondrial buffer (2 mL for 200 mg of tissue) supplemented with trypsin (0.5 mg/g of tissue) was obtained using a glass grinder (TissueRuptor II, Qiagen, Les Ulis, France). The suspension was then centrifuged at 700 × g for 10 min at 4°C to pellet cell debris, and the supernatant was further centrifuged at 7000 × g for 10 min at 4°C to pellet mitochondria. Pelleted mitochondria were then suspended in a mitochondrial buffer (30 to 50 μL) to obtain the mitochondrial enriched fraction. Protein concentration was then determined using the Bradford assay, with one part of the mitochondrial enriched fraction then immediately analyzed for mitochondrial respiration using an Oxygraph 2K (Oroboros Inc., Innsbruck, Austria), and the remaining frozen on dry ice.

### 2.4 Mitochondrial Respiration Protocols

High‐resolution tissue respirometry (HRR) of permeabilized muscle fibers offers an integrative ex vivo measure of the dynamics of coupled metabolic pathways. HRR of isolated mitochondria provides respiratory rates of the mitochondria per second.

Quantification of oxygen consumption in both permeabilized fiber bundles and isolated mitochondria was conducted in an ambient oxygen concentration of about 180 nmol O_2_/mL over a ~2 h period. Experiments with permeabilized fiber bundles were run at both 37°C and 33°C for each sample to mimic the body temperature of the active and hibernating animals in summer and winter, respectively, based on previous measurements in this population [23]. Because we only had three respirometers on site, experiments with isolated mitochondria were run once at 25°C. We chose this non‐physiological temperature to remove any potential interaction between the ambient temperature and the season on the mitochondrial respiratory rates.

In all experiments, substrate–uncoupler–inhibitor titration (SUIT) protocols for carbohydrates (CHO) and fatty acid (FAT) respiration were performed using an Oxygraph‐2K (O2K) respirometer (Oroboros Instruments, Innsbruck, Austria) and following the stabilization of the O_2_ trace (Table 1). The addition of cytochrome *c* was used to confirm the integrity of the outer mitochondrial membrane and the quality control threshold was set at ≤ 15% increase in respiration. We assessed oxygen consumption ex vivo in primary respiratory states, including measurement of LEAK respiration, OXPHOS, and MAX respiratory flux through respiratory complexes I and II (CI + CII). Uncoupled MAX respiration reflects the maximum mitochondrial respiratory flux [24]. OXPHOS respiratory capacity represents the portion of MAX capacity composed of both the respiratory flux coupled to mitochondrial oxidative phosphorylation through ATP synthase and non‐coupled LEAK respiration. Steady‐state respiratory values were normalized to fiber bundle wet weight and corrected for baseline respiratory chamber oxygen flux determined at the beginning of each protocol. Oxygen flux was monitored in real‐time following standardized instrumental and chemical background calibrations using Datlab 5.0. software (Oroboros Instruments).

**TABLE 1:** High‐resolution respirometry protocols and associated respiratory states for muscle fiber mitochondrial respiration experiments.

Protocol components (in order of titration)		Respiratory state
FAT SUIT	Palmitoyl‐carnitine (0.01 mM) + malate (0.8 mM)	LEAK; uncoupled respiration in the presence of long and short chain fatty acid concentration
	Octanoyl‐carnitine (0.1 M)	
	ADP (5 mM)	OXPHOS (CI); Fatty acid stimulated respiration
	Pyruvate (2 M) + Glutamate (2 M) + Succinate (1 M)	OXPHOS (CI + CII); Complex I + II coupled respiration
	Carbonylcyanide *p*‐trifluoromethoxy‐phenylhydrazone (FCCP, 1 mM)	ETS (CI + CII); Maximal uncoupled Complex I and II capacity
	Rotenone (1 mM)	ETS (CII); Maximum uncoupled Complex II capacity
	Malonate (2 M) + Antimycine (5 mM)	ROX; Residual oxygen concentration
CHO SUIT	Pyruvate (2 M) + Malate (0.8 M)	LEAK; Complex I uncoupled respiration
	Glutamate (2 M)	
	ADP (0.5 M)	OXPHOS (CI); Complex I coupled respiration
	Cytochrome c (4 mM)	CytC; Test of outer mitochondrial membrane integrity
	Succinate (1 M)	OXPHOS (CI + CII); Complex I + II coupled respiration
	Carbonylcyanide *p*‐trifluoromethoxy‐phenylhydrazone (FCCP, 1 mM)	ETS (CI + CII); Maximal uncoupled Complex I and II capacity
	Rotenone (1 mM)	ETS (CII); Maximum uncoupled Complex II capacity
	Malonate (2 M) + Antimycine (5 mM)	ROX; Residual oxygen concentration

In the CHO SUIT, LEAK state without adenylates respiration (i.e., substrate‐driven non‐phosphorylating inner membrane proton leak) was determined by the addition of saturating concentrations of pyruvate (2 M) and malate (0.8 M), and subsequently of glutamate (2 M). Oxidative phosphorylation (OXPHOS) by electron flux through complex I was measured with the addition of ADP (0.5 M). Cytochrome c (4 mM) was then added to verify mitochondrial outer membrane integrity. OXPHOS through complex I (CI) and complex II (CII) was measured with the addition of succinate (1 M). Titrations of the uncoupler FCCP (carbonyl cyanide‐p‐trifluoromethoxyphenylhydrazone, 1 mM) were performed to assess maximal electron transport system (ETS CI + CII) respiration. Lastly, complex I inhibitor rotenone (1 mM) was added to measure the rate of respiration through complex II alone (ETS CII). To finish, malonic acid (2 M) and antimycin A (5 mM) were added to inhibit complex II and complex III, respectively. With total electron transport chain respiration inhibition, residual oxygen consumption (ROX) was then quantified. Because ROX was often higher than LEAK, we did not use it as a correction factor for all mitochondrial respiratory rates as commonly done; ROX values are shown in Figure 7.

In the FAT SUIT, LEAK state without adenylates respiration was measured by the addition of palmitoyl‐carnitine (0.01 mM), octanoylcarnitine (0.1 M) and malate (0.8 mM). This was followed by ADP (0.5 mM) for State 3 coupled respiration and then pyruvate (2 M), glutamate (2 M) and succinate (1 M) for OXPHOS. The remainder of the FAT SUIT protocol was the same as the CHO SUIT.

For both substrate‐specific SUIT protocols, the respiratory control ratio (RCR) was calculated (State 3/LEAK) to determine the degree of coupled respiration and the flux control ratio (FCR; each respiratory state normalized to maximal respiratory rate, that is, ETS CI + CII) to determine oxygen flux in different respiratory control states.

### 2.5 Proteomics of Bear Muscle and Mitochondrial Enriched Fractions

The process of sample preparation, nanoLC‐MS/MS analysis, and mass spectrometry data analysis is described in detail in Supporting Information. The procedure involved protein extraction and electrophoresis using SDS‐PAGE, followed by in‐gel digestion with trypsin (Promega, Madison, WI, USA). The resulting peptides were extracted and analyzed using a nanoUPLC‐system (nano‐Acquity, Waters, Milford, MA, USA) coupled to a quadrupole‐Orbitrap hybrid mass spectrometer (Q‐Exactive HF‐X for total proteins and Q‐Exactive plus for mitochondrial proteins, Thermo Scientific, San Jose, CA, USA). Mass spectrometers were operated using a data‐dependent acquisition strategy by selecting the Top‐20 (HF‐X) or Top‐10 (Plus) most intense ions in MS1 for fragmentation in MS2. MS raw data processing was performed in MaxQuant (v1.6.11.0 or v2.6.3.0) [25], using a protein database containing RefSeq sequences for *Ursus arctos* proteins (GCF_003584765.1 and GCF_023065955.2 assemblies). The mass spectrometry proteomics data have been deposited to the ProteomeXchange Consortium via the PRIDE [26] partner repository with the dataset identifiers PXD060503 for the total protein experiment and PXD036916 for the mitochondrial experiment. Further details can be found in Supporting Information Material and Method.

### 2.6 Western‐Blot Analysis and SDH Enzymatic Activity

Western‐blot analysis was performed from frozen mitochondrial enriched fractions and from a whole‐muscle lysate obtained as described previously [27]. After denaturation in Laemmli buffer at 95°C for 5 min, 30 μg of muscle lysate and 5 μg from mitochondrial suspension were loaded on precast gels (Mini‐protean TGX Stain‐free gel, Biorad, Hercules, CA, USA). Electrophoresis was followed by UV exposure for 3 min, and gels were imaged for further quantification of protein loading using G:BOX ChemiXT4 (XL1) imaging system (Syngene, Frederick, MD, USA). After a semi‐dry transfer onto PVDF membranes (Hybond P, Amersham, England), membranes were blocked with 4% BSA (Bovine Serum Albumin, Euromedex, Souffelweyersheim, France) before incubation with primary antibodies against citrate synthase (sc‐390693, SCBT) and OXPHOS complexes (ab110413, Abcam) respectively. An anti‐goat secondary horseradish peroxidase (HRP) antibody was used for chemiluminescence revelation using G:BOX ChemiXT4 (XL1) imaging system (Syngene, Frederick, MD, USA). Signals were then quantified using ImageJ software and normalized to the loaded amount of proteins determined by TGX intensity.

Protein extracts from muscle lysates and mitochondrial suspensions were also used to quantify succinate dehydrogenase (SDH) activity using a commercial kit (#MAK197) from Sigma‐Aldrich (St. Louis, MO, USA). SDH activity was calculated as the amount of substrate converted per minute and normalized to the amount of protein loaded of each sample and is expressed as nmol·min^−1^·μg^−1^ protein.

### 2.7 Mitochondrial Content in Bear Muscles

To quantify the mitochondrial DNA content in bear muscle samples, two DNA regions were selected and amplified: one for mitochondrial DNA and one for nuclear DNA (primers Ua‐mtdna‐S1: 5′‐ATTT) where *R* is the mtDNA/nucDNA ratio, *E*_mt_ the PCR efficiency of the mitochondrial target, and *E*_nuc_ the PCR efficiency of a nuclear target. *ΔCt*_mt_ and *ΔCt*_nuc_ were calculated as the difference of Ct from the mean of all samples for the corresponding amplified regions.

### 2.8 Skeletal Muscle Fiber Type

Muscle cross‐sections (10 μm thick) were obtained using a cryostat (HM500M Microm International, Fisher Scientific, Illkirch, France) at −20°C. Subsequently, the sections were incubated at room temperature for 1 h in a blocking buffer (10% Goat serum in PBS). Cross‐sections were labeled with anti‐laminin‐α1 (L9393 Sigma, 1/200, Saint‐Quentin‐Fallavier, France) to outline the fibers and anti‐MyH1 antibody (BAD5, 1/50, Developmental Studies Hybridoma Bank (DSHB) at the University of Iowa) to detect type I fibers. Prior to the analyses, we validated the specificity of commercially available antibodies against MyHC isoforms using a combination of ATPase staining and immunohistochemistry. Only the anti‐MyH1 (BAD5) antibody reliably labeled Type I (slow‐oxidative) fibers in bear skeletal muscle. Antibodies commonly used to distinguish Type IIa (fast oxidative–glycolytic) and Type IIb (fast glycolytic) fibers in rodents did not yield specific or consistent staining in bears. Although a small subset of fibers showed stronger labeling consistent with a IIb phenotype, this distinction was not sufficiently reproducible. For these reasons, all non‐labeled fibers were classified as Type II for the purposes of this study. The primary antibody diluent buffer contained 10% goat serum. Each antibody was loaded onto a specimen and incubated overnight at 4°C. After washing with PBS, muscle cross‐sections were resolved with corresponding secondary antibodies conjugated to Alexa‐Fluor 488 for laminin, and Alexa‐Fluor 546 for MyH1 (Invitrogen, Cergy‐Pontoise, France) for 1 h at room temperature. Coverslips were then mounted on the samples with Fluorescent Mounting Medium (Shandon Immu‐Mount Ref 9990402 Thermo Scientific). Observations and image acquisitions were captured with a high‐resolutionORCA‐Flash4.0 LT+ Digital CMOS camera coupled to a IX‐73 microscope (Olympus, Plan Fluorite Long Working Distance Objective 20X Phase—LUCPLFLN20XPH, Münster, Germany) and Cell‐Sens dimension software (Olympus Soft Imaging Solutions, Münster, Germany). From 950 to 3500 fibers were analyzed in all samples.

### 2.9 Data and Statistical Analyses

The number of samples varies across assays because the muscle biopsy material obtained for each bear was limited, and not all downstream analyses could be performed on every animal after completing the mitochondrial respiration experiments. Sample size is indicated in the legends of all figures.

Data normality and homoscedasticity were assessed using the Shapiro–Wilk and Bartlett tests, respectively (*p* > 0.01). When necessary, data were log‐transformed to meet assumptions. Changes in bear body mass, mitochondrial respiratory rates (in isolated mitochondria), western blot data, and succinate dehydrogenase (SDH) activity between hibernating and summer‐active bears were analyzed using paired Student's *t*‐tests, with significance set at *p* < 0.05. For proteomics experiments, changes were analyzed using paired Student's *t*‐tests, but also using Welch's two‐sample *t*‐tests due to missing values, with significance set at *p* < 0.05.

Linear mixed models (LMM) were used to test for differences in respiratory states of permeabilized muscle fibers (LEAK, OXPHOS (CompI), OXPHOS (CI + CII), ETS (CI + CII), and ETS (CII)) and their respective ratios. The models included season, temperature, sex, body mass and the season‐by‐temperature interaction as fixed effects and animals as random effects. Because sex and body mass had no effect on the primary outcomes and did not alter the significance of seasonal or temperature effects, they were removed to retain the most parsimonious model. Results presented in the manuscript therefore derive from LMMs including only season, temperature and their interaction as fixed effects. Changes in proportion of muscle fiber type and fiber areas were analyzed using LMM following the same approach and retained with season as fixed effect and animals as random effect.

The optimal covariance structure was selected based on Akaike Information Criterion (AIC) and Bayesian Information Criterion (BIC) values. Model interactions were considered significant at *p* ≤ 0.1. Tukey's post hoc test was applied to examine specific differences between interventions, with *p* ≤ 0.05 indicating significance.

To evaluate the relative contributions of carbohydrates and fatty acids to mitochondrial respiration, an additional LMM was used with season, temperature, substrate type, and their interactions (season × temperature, season × substrate, temperature × substrate, and the three‐way interaction season × temperature × substrate) as fixed effects and animals as random effects. Given the small sample sizes, no post hoc tests were performed following this model.

All analyses were conducted using SAS software, version 9.2 (SAS Institute, North Carolina, USA) and GraphPad Prism version 10.2.3. Data are presented as mean ± SD unless otherwise specified.

## 3 Results

### 3.1 Hibernation Reduces Mitochondrial Proteomic Fraction but Preserves Protein Composition and Muscle Fiber Type

**FIGURE 1:**
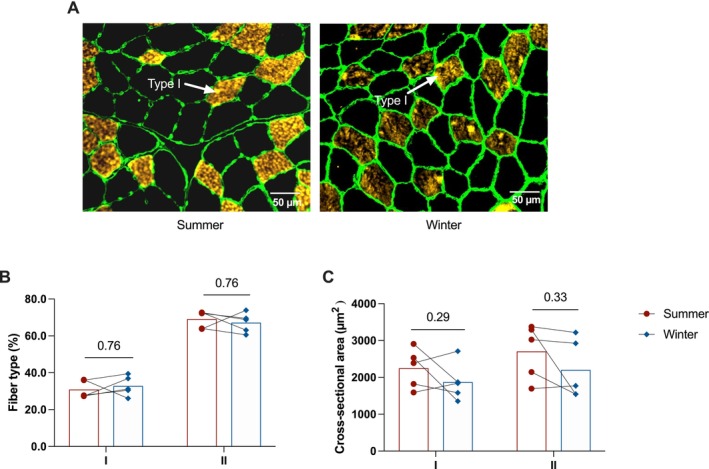
Muscle fiber type in skeletal muscle biopsies collected from active and hibernating brown bears. Representative immunofluorescence images of muscle cross‐sections showing type I fibers (yellow/orange) and cell membranes (green) from the same animal in summer (*n* = 5, summer) and winter (*n* = 5, winter) are presented in A. The percentage of type I and type II fibers quantified in skeletal muscle biopsies from the same animals is shown in B, and the cross‐sectional area of type I and type II fibers is shown in C. Data are expressed as the mean with individual datapoints represented by dots (summer) and diamonds (winter), Red bars, summer; blue bars, winter.

**FIGURE 2:**
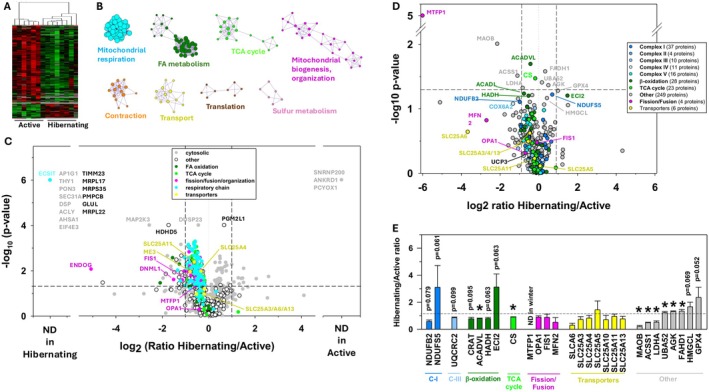
Proteomic changes in bear skeletal muscle during hibernation (winter) compared with the summer‐active period. Overview of differentially‐expressed proteins (*p* < 0.05) in skeletal muscle from hibernating (*n* = 7, winter) compared with physically active (*n* = 7, summer) bears (A, hierarchical clustering with down‐regulated proteins in green and up‐regulated ones in red). Functional annotation enrichment analysis from differentially‐expressed muscle proteins, highlighting the main biological processes differentially affected during hibernation (B). Seasonal changes in muscle protein abundances are illustrated as a volcano plot, highlighting categorized proteins using colors (C, vertical, and horizontal dashed lines show the thresholds we used for *p*‐values and protein abundance ratios between seasons). Seasonal changes of muscle protein abundances in the mitochondrial enriched fraction prepared from skeletal muscle of hibernating (*n* = 4, winter) and physically active (*n* = 4, summer) bears, illustrated as a volcano plot to highlight categorized proteins using colors (D, vertical and horizontal dashed lines show the thresholds we used for *p*‐values and protein abundance ratios between seasons). Abundance ratios (calculated by considering paired values obtained during both seasons) for the few differentially expressed proteins (*, *p* < 0.05) in mitochondrial‐enriched fractions and for those with a *p*‐value close to significance (E, abundance changes in mitochondrial proteins). Detailed protein abundances and fold changes are given in Table S2 (skeletal muscle proteome) and 3 (skeletal muscle mitochondrial fraction proteome). FA, fatty acids; TCA, tricarboxylic acid cycle.

No change in skeletal muscle fiber type (*p* = 0.76; Figure 1B) nor in the cross‐sectional area (type I: *p* = 0.29; type II: *p* = 0.33; Figure 1C) was noted in winter compared to summer. Using stringent criteria established for mass spectrometry data validation (see M&M), 1705 proteins were identified from bear muscle extracts, and 1335 of them were retained for quantification (Table S2). Among these 1335 proteins, 357 were found to be annotated as being mitochondrial in Uniprot and/or Nextprot knowledge bases (i.e., 26.7%). Statistical analysis highlighted 284 proteins whose abundance changed with a *p*‐value lower than 0.05 (Welsh's *t*‐test). Three additional proteins were only detected in winter samples and 15 others only in summer samples, presumably due to seasonally low abundances (i.e., they were below our lower limit of detection) (Figure 2A). Among these 302 differentially expressed muscle proteins, 191 were mitochondrial proteins (i.e., 63.2%), underlining the central role of mitochondria in response to hypometabolism during hibernation. Functional annotation enrichment analysis from the set of differentially abundant proteins further reinforced this observation by enabling us to highlight eight significantly enriched processes, that is, processes that are particularly affected by hibernation (Figure 2B). Among these, mitochondrial processes included the respiratory function (nearly all subunits of the five mitochondrial respiratory complexes were quantified), metabolism of fatty acids, TCA, mitochondrial biogenesis and organization, and molecular transport. The other affected processes include contraction, protein translation, and sulfur metabolism. The variation in abundance is illustrated for differential proteins in these various processes (Figure 2C), showing the predominance of downregulation for mitochondrial processes such as respiration, fatty acid oxidation, and the TCA.

When we analyzed bear mitochondria extracts, 967 proteins were identified, with 720 retained for quantification (Table S3). Among these 720 proteins, 328 were found to be annotated as being mitochondrial proteins in Uniprot and/or Nextprot knowledge bases (i.e., 45.6%). Hence, these results show that the sample preparation of mitochondrial extracts resulted in mitochondria enrichment, given its higher mitochondrial concentration when compared to the skeletal muscle lysate (26.7% mitochondrial). Statistical analysis from the mitochondrial extract experiment highlighted only 18 proteins with a *p*‐value lower than 0.05 (paired *t*‐test), with three additional proteins that were only detected in summer samples (see variations for all mitochondrial proteins in Figure 2D). Of these 21 differential proteins, eight were identified to be mitochondrial. Eleven additional proteins exhibited a *p*‐value higher than 0.05 but lower than 0.07 (paired *t*‐test), eight of which were mitochondrial. The abundance of 26 important mitochondrial proteins is shown in Figure 2E. These results strongly support the idea that the mitochondrial responses observed in hibernating bear muscle arise primarily from a reduction in overall mitochondrial content within the cells, rather than from major alterations to the molecular machinery of the mitochondria themselves. In other words, the core components that regulate mitochondrial dynamics (OPA1, FIS1, MFN1), substrate transport, and the electron transport system (Complex I and Complex III) remain largely stable across seasons. This indicates that the mitochondria that are retained during hibernation maintain a broadly similar functional and structural profile. The seasonal differences observed in specific metabolic enzymes (ACADVL, CS, MAOB, ACSS1, LDHA, UBA52, AGK, FADH1) therefore likely reflect fine‐tuned adjustments in metabolic flux or substrate preference, rather than a wholesale remodeling of mitochondrial architecture or composition.

### 3.2 Reduced Mitochondrial Oxidative Capacity Extends Beyond Decreased Mitochondrial Density During Hibernation

**FIGURE 3:**
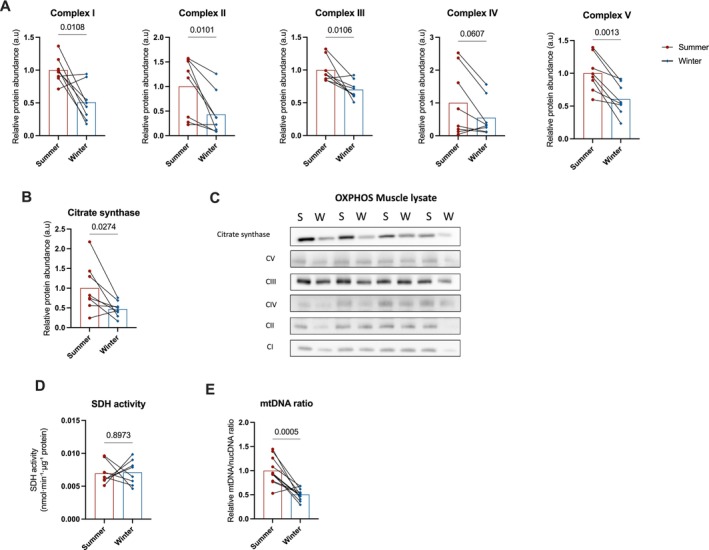
Protein expression of the complexes of the electron transport chain and citrate synthase, and succinate dehydrogenase activity measured in the whole skeletal muscle lysate collected in active and hibernating brown bears. The protein expression of complexes I–V of the electron transport chain, assessed via a single representative peptide subunit for each complex, located in the inner mitochondrial membrane (A) and of citrate synthase (B) were measured in skeletal muscle biopsies collected in summer physically active (*n* = 8, summer) and winter hibernating bears (*n* = 8, winter). All protein expression was quantified using western blot with densitometry represented in C. Protein expression is expressed as raw data normalized to loaded protein. The activity of the enzyme succinate dehydrogenase (SDH) (D) and mitochondrial DNA/RNA ratio per gram of muscle (E) were also measured in 8 animals. Differences between summer and winter were statistically analyzed using a paired *T*‐test. Data are expressed as the mean with the individual data points represented by dots (summer) and diamonds (winter). Red bars, summer; blue bars, winter.

**FIGURE 4:**
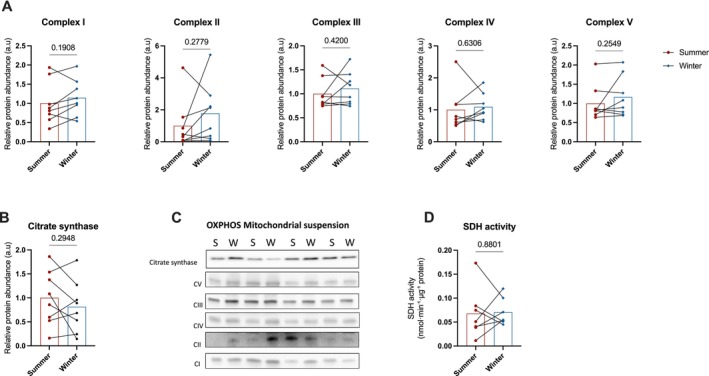
Protein expression of the complexes of the electron transport chain and citrate synthase, and succinate dehydrogenase activity measured in the mitochondria isolated from skeletal muscle biopsy collected in active and hibernating brown bears. The protein expression of complexes I–V of the electron transport chain located in the outer mitochondrial membrane (A) and of citrate synthase (B) were measured in mitochondria isolated from skeletal muscle biopsies collected in summer physically active (*n* = 8, summer) and winter hibernating bears (*n* = 8, winter). Protein expression is expressed as raw data normalized for the loaded amount of protein. All protein expression was quantified using a western blot with densitometry represented in C. The activity of the enzyme succinate dehydrogenase (SDH, D) was also measured in 7 animals. Differences between summer and winter were statistically analyzed using a paired *t*‐test. Data are expressed as the mean with the individual datapoints represented by dots (summer) and diamonds (winter). Red bars, summer; blue bars, winter.

Given the observed proteomic changes reflecting the reduction in mitochondrial content, we next assessed mitochondrial abundance and energetics more directly. We assessed the expression of key subunits of mitochondrial respiratory chain complexes (Figure 3A,B) and citrate synthase (Figure 3B) by Western blot in bear muscle extracts. All protein levels were significantly lower in winter compared to summer muscle, consistent with our mass spectrometry‐based proteomics data that revealed a significant reduction in the abundance of these proteins in hibernating bear muscle (*p* < 0.03 for all, except *p* = 0.061 for MT‐CO1, a subunit of Complex IV). As already shown through our proteomic analysis (see above), these changes were accompanied by a marked reduction in the mitochondrial‐to‐nuclear DNA ratio per gram of muscle (*p* = 0.0005, Figure 3E), indicating a decreased mitochondrial content during hibernation. When examining isolated mitochondria, no significant seasonal differences were observed in the expression of respiratory complex subunits or citrate synthase (Figure 4A–C), suggesting that the protein composition within mitochondria remains relatively stable.

Interestingly, despite the decline in the abundance of SDH (Complex II) in whole muscle (Figure 3A), its activity remained unchanged between seasons, both in skeletal muscle lysates (Figure 3D) and isolated mitochondria (Figure 4D). This preservation of SDH activity may play a key role in maintaining mitochondrial respiratory capacity at low temperatures during hibernation.

### 3.3 Hibernation Reduces Skeletal Muscle Fiber Coupled Respiration in the Presence of Carbohydrate‐Like Substrate, Independent of Reduced Mitochondrial Density

**FIGURE 5:**
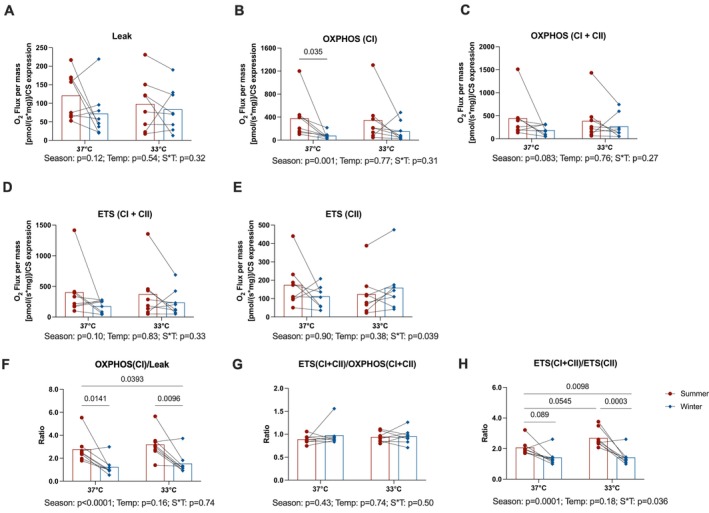
Mitochondrial respiration in permeabilized muscle fibers in the presence of carbohydrate‐like substrates measured in active and hibernating brown bears. Mitochondrial respiration normalized for tissue wet weight and citrate synthase (CS) expression in the presence of carbohydrate (CHO SUIT) supported substrates in skeletal muscle permeabilized fibers collected from summer physically active (*n* = 8, summer) and winter hibernating bears (*n* = 8, winter). High‐resolution respirometry experiments were used to measure LEAK respiration in the presence of pyruvate and malate (panel A), ADP‐coupled respiration through Complex I (OXPHOS (CI), panel B); ADP‐coupled respiration through Complex I and Complex II (OXPHOS (CI + CII), panel C), maximal respiration through Complex I and Complex II (ETS (CI + CII), panel D), respiratory capacity through Complex II only when Complex I is inhibited (ETS (CII), panel E) were measured at both 33°C and 37°C. Ratios between coupled (OXPHOS (CI)) and uncoupled (LEAK) respiration (panel F), between maximal respiration through Complex I and Complex II (ETS (CI + CII)) and coupled respiration (OXPHOS (CI + CII)) (panel G), and between maximal respiration through Complex I and Complex II (ETS (CI + CII)) and respiratory capacity through Complex II only when Complex I is inhibited (ETS (CII)) (panel H) are also presented. Season, Season effect; Temp, Temperature effect; S*T, season‐by‐temperature interaction. Statistical analysis are the results of the linear mixed model followed by Tukey post hoc tests. Data are expressed as the mean ± SEM, Red bars, summer; blue bars, winter. Individual response represented by dots (summer) and diamonds (winter).

To determine whether changes in protein expression corresponded to functional impairments, we examined mitochondrial respiratory capacity in permeabilized muscle fibers and isolated mitochondria. Respiratory rates within the permeabilized muscle fibers (normalized to muscle fiber weight) were significantly lower in winter than in summer, regardless of the experimental temperature (Season effect: *p* < 0.006 for all, Table S4). After correcting these rates for the seasonal decrease in citrate synthase abundance (−16%, *p* = 0.027, Figure 2B and Table S4), uncoupled (Figure 5A) and maximal ETS cellular respiration (Figure 5D,E) were no longer significantly different between summer and winter. However, coupled respiration remained lower in hibernating bears during winter compared to active bears in summer, especially under conditions where only complex I was activated (OXPHOS CI: LSmeans (SE) −0.176 (0.109) pmol(s)/citrate synthase expression, Season effect: *p* = 0.001, Figure 5B; OXPHOS (CI + CII): Season effect: *p* = 0.083, Figure 5C). The ratio of coupled to uncoupled respiration was also significantly reduced during hibernation (Figure 5F, Season effect: *p* = 0.0045), suggesting a marked drop in the ADP‐driven respiration to a level barely above the basal substrate‐driven LEAK respiration in hibernating bears.

In the isolated mitochondria, all the respiratory rates measured in the presence of carbohydrate‐like substrates at 25°C were reduced or tended to be reduced with hibernation (*p* < 0.059 for all, Table S5). When correcting for the changes in mitochondrial protein content, the basal, non‐ADP‐driven respiration rate (LEAK, *p* = 0.026) and the maximal ETS respiration relying on complex II (*p* = 0.034) were still significantly lower in winter compared to summer. Oxidative phosphorylation supported either solely by complex I (OXPHOS (CI): *p* = 0.090) or by both complex I and complex II (OXPHOS (CI + CII): *p* = 0.10), and the maximal electron transport system capacity driven by both complexes (ETS (CI + CII): *p* = 0.053) also tended to be lower in winter compared to summer (Figure S1A). Similarly, the uncoupled, coupled, and maximal respiratory rates corrected for mitochondrial content were lower in winter compared to summer with fatty acid‐carlike substrates (*p* < 0.07 for all, Figure S1B and Table S5). Altogether, these data indicate a trend toward a reduced mitochondrial oxidative capacity during hibernation that is exacerbated by the decrease in mitochondrial density.

The ratios between maximal ETS capacity and coupled respiration (i.e., ETS (CI + CII)/OXPHOS (CI + CII)), measured in both the permeabilized fibers (Figure 5G) and isolated mitochondria (Figure S1B), were not affected by the season, indicating that in both summer and winter, mitochondria have the capability to operate at near maximal capacity.

### 3.4 Hibernating Skeletal Muscle Is Resilient to Temperature‐Dependent Declines in Mitochondrial Respiration

In permeabilized skeletal muscle fibers, respiratory rates assessed using carbohydrate‐supported substrates (Figure 5A–D) were largely unaffected by experimental temperature (33°C vs. 37°C), suggesting a minimal temperature effect. Notably, maximal ETS cellular respiration through complex II alone was significantly higher in winter tested at 33°C compared to both summer muscle at 33°C and winter muscle at 37°C (Season*Temperature interaction: *p* = 0.039, Figure 5E). This suggests that skeletal muscle during hibernation may be biochemically programmed to maintain mitochondrial energetics in the presence of carbohydrates at lower body temperatures.

**FIGURE 6:**
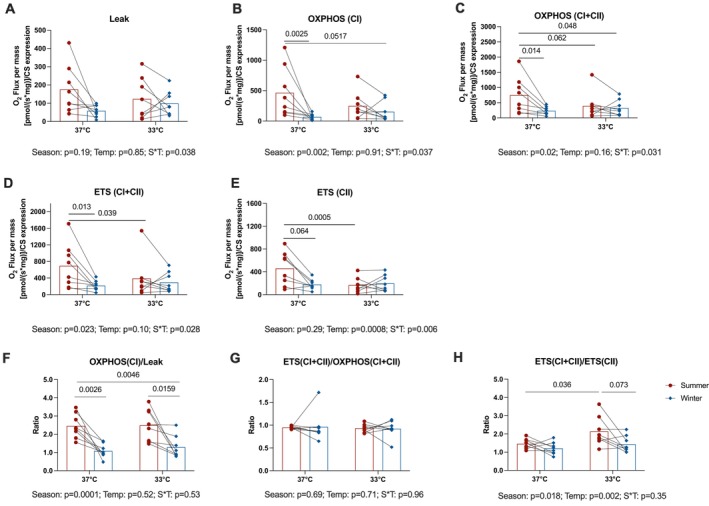
Mitochondrial respiration in permeabilized muscle fibers in the presence of fatty acid‐supported substrates measured in active and hibernating brown bears. Mitochondrial respiration normalized for tissue wet weight and citrate synthase (CS) expression in the presence of fatty acids (FAT SUIT) supported substrates in skeletal muscle permeabilized fibers collected from summer physically active (*n* = 8, summer) and winter hibernating bears (*n* = 8, winter). High‐resolution respirometry experiments were used to measure LEAK respiration in the presence of pyruvate, malate and octanoylcarnitine (panel A), ADP‐coupled respiration through Complex I (OXPHOS (CI), panel B); ADP‐coupled respiration through Complex I and Complex II (OXPHOS (CI + CII), panel C), maximal respiration through Complex I and Complex II (ETS (CI + CII), panel D), respiratory capacity through Complex II only when Complex I is inhibited (ETS (CII), panel E) were measured at both 33°C and 37°C. Data for the ratios between coupled (OXPHOS (CI)) and uncoupled (LEAK) respiration (panel F), between maximal respiration through Complex I and Complex II (ETS (CI + CII)) and coupled respiration (OXPHOS (CI + CII)) (panel G), and between maximal respiration through Complex I and Complex II (ETS (CI + CII)) and respiratory capacity through Complex II only when Complex I is inhibited (ETS (CII)) (panel H) are also presented. Season, Season effect; Temp, Temperature effect; S × T, season‐by‐temperature interaction. Statistical analyses are the results of the linear mixed model followed by Tukey's post hoc tests. Data are expressed as the mean ± SEM. Red bars, summer; blue bars, winter. Individual responses represented by dots (summer) and diamonds (winter).

In contrast, in the presence of fatty acid‐supported substrates, experimental temperature strongly influenced respiratory rates. Uncoupled, coupled, and maximal respiratory rates normalized to citrate synthase protein abundance were all reduced during hibernation—but only when assays were performed at 37°C, not at 33°C (Season*Temperature interaction: *p* < 0.038 for all, Figure 6A–E). This pattern arose because lower experimental temperature reduced respiratory rates in muscles from active (summer) bears but did not impact the respiratory rates in muscle mitochondria from hibernating (winter) bears.

### 3.5 Shift Toward Increased Reliance on Complex‐II Respiration During Hibernation

The ratio of ETS (CI + CII) to ETS (CII) was significantly reduced during hibernation (*p* = 0.0001; Figure 5H), with winter muscle tested at 33°C exhibiting a lower ratio than summer muscle tested at 37°C (*p* = 0.0098). This suggests that, following inhibition of Complex I by rotenone, skeletal muscle from hibernating bears may exhibit increased Complex II‐mediated respiration when provided with a saturating concentration of succinate ex vivo. A similar capacity has been observed in small hibernators, such as thirteen‐lined ground squirrels ( *Ictidomys tridecemlineatus* ), where succinate‐driven respiration is retained during torpor despite suppression of NADH‐linked pathways [28]. Functionally, this preserved Complex II capacity may help maintain ATP production and mitochondrial energetics under low‐temperature, low‐metabolic‐demand conditions, such as those encountered during hibernation, providing a metabolic support system when NADH‐linked (Complex I) substrates are limited.

Similarly, when fatty acid‐supported substrates were used, although no significant Season*Temperature interaction was observed, the ratio of ETS (CI + CII) to ETS (CII) was also significantly reduced during hibernation (Season: *p* = 0.018) and was lower at 37°C compared to 33°C (Temperature: *p* = 0.002, Figure 6H).

Together, these findings demonstrate that hibernation is associated with a consistent shift toward increased reliance on Complex II (SDH), irrespective of the substrate type. Furthermore, lower temperatures appear to amplify this shift, suggesting a temperature‐dependent response that preserves mitochondrial electron transport through Complex II while minimizing Complex I contribution during hibernation.

### 3.6 Winter Muscles Have Lower Ex Vivo Mitochondrial Maximal Cellular Respiration Across Substrates

**FIGURE 7:**
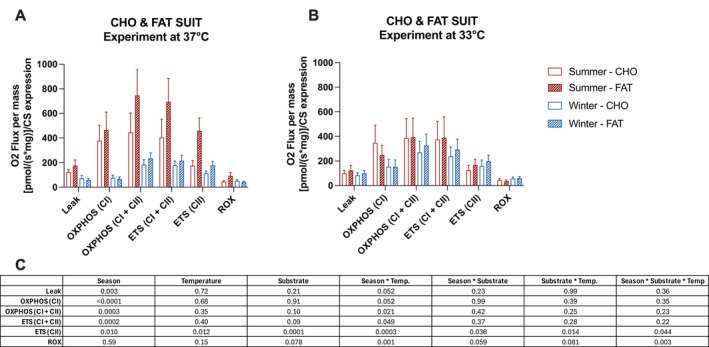
Contribution of carbohydrate and fatty acid‐like substrates to mitochondrial respiratory rates at 37°C and 33°C measured in active and hibernating brown bears. Mitochondrial respiration normalized for tissue wet weight and citrate synthase (CS) expression in the presence of either carbohydrate (CHO SUIT) or fatty acid (FAT SUIT) associated substrates in skeletal muscle permeabilized fibers collected from summer physically active (*n* = 8, summer) and winter hibernating bears (*n* = 8, winter) when the experiments were run at either 37°C (A) or 33°C (B). Respiratory rates were measured for LEAK respiration in the presence of pyruvate and malate in the CHO SUIT and with pyruvate, malate, and octanoylcarnitine for the FAT SUIT, ADP‐coupled respiration through Complex I (OXPHOS (CI)); ADP‐coupled respiration through Complex I and Complex II (OXPHOS (CI + CII)), maximal respiration through Complex I and Complex II (ETS (CI + CII)), respiratory capacity through Complex II only when Complex I is inhibited (ETS (CII)). These data are the same as those presented in Figures 1 and 2. Panel C presents the *p*‐values generated by the analyses with linear mixed models with fixed effects being season, temperature, substrate, season‐by‐substrate interaction, temperature‐by‐substrate interaction, season‐by‐temperature‐by‐substrate interaction, and random effect being animals. S × T × Subs, season‐by‐temperature‐by substrate interaction; S, season effect; Subs, substrate effect; T, temperature effect. Data are expressed as the mean ± SEM, Red bars, summer; blue bars, winter. Individual response represented by dots (summer) and diamonds (winter).

Previous studies have shown that lipid substrates remain the primary fuel source to provide energy during hibernation in bears, likely due to the inhibition of pyruvate dehydrogenase activity by PDK4 kinase in skeletal muscle. However, it has been hypothesized that carbohydrate metabolism may still be maintained to support rapid energy demands during unexpected arousals [15]. To explore this possibility, we compared mitochondrial respiratory rates in permeabilized muscle fibers in the presence of either carbohydrate‐ or fatty acid‐supported substrates, measured at both 33°C and 37°C. The data presented in Figure 7 are the same as those shown in Figures 5 and 6 but reorganized side‐by‐side to better examine potential seasonal and temperature‐dependent differences in substrate utilization. Statistical analyses were conducted to determine whether skeletal muscle exhibits preferential substrate use depending on season or temperature.

When experiments were run at 33°C, no significant differences in respiratory rates using carbohydrate‐ or fatty acid‐supported substrates were observed either in summer or winter muscles (Figure 7A,B). However, at 37°C, a significant three‐way interaction between season, temperature, and substrate was detected for maximal respiration through Complex II (ETS CII, *p* = 0.044). Specifically, mitochondrial respiration was greater in the presence of fatty acid‐supported substrates compared to carbohydrate‐supported substrates in the skeletal muscle of active (summer) bears at 37°C. This indicates that skeletal muscle from active bears preferentially oxidizes fatty acids over carbohydrates at normothermia, whereas such substrate preference is not evident at hibernation temperature.

## 4 Discussion

By comparing mitochondrial respiratory rates in isolated mitochondria and permeabilized skeletal muscle fibers between summer and winter seasons, this study revealed that hibernating brown bears preserve skeletal muscle mitochondrial energetics through a coordinated set of protective strategies. Despite a seasonal reduction in mitochondrial density, mitochondrial energetics remain largely intact. Specifically, we observed in winter, compared to summer, (1) reduced respiration rates, primarily driven by lower mitochondrial density rather than intrinsic dysfunction; (2) preserved coupled respiration with carbohydrate‐supported substrates; (3) a shift toward Complex II reliance for mitochondrial respiration, indicating preserved SDH function under hypometabolic conditions, likely enhanced by lower physiological temperatures; and (4) retained flexibility to oxidize both fatty acids and carbohydrates ex vivo. Together, these responses ensure that skeletal muscle remains functional, flexible in substrate use, and energy‐efficient during hibernation, while capable of rapid metabolic reactivation during arousal events.

### 4.1 Hibernating Brown Bears Preserve Skeletal Muscle Fiber Types, Mitochondrial Machinery and Energetics

Our findings show that the systemic responses during hibernation (e.g., decreases in whole‐body metabolic rates) are mirrored at the muscular level, where OXPHOS capacity is markedly reduced. This response is essentially due to lower mitochondrial content, which likely results from the prolonged physical inactivity that bears experience during the 5 to 7 months of hibernation. Similar mitochondrial molecular responses have been reported in the muscle of hibernating Asiatic bears [29]; yet, to our knowledge, this is the first study to comprehensively assess mitochondrial energetics along with the underlying regulatory mechanisms in the skeletal muscle of a hibernating mammal.

Despite the reduction in mitochondrial density, proteomic analysis of isolated mitochondria revealed a preservation of core mitochondrial machinery, including Krebs cycle enzymes. While protein expression of citrate synthase and other mitochondrial markers was reduced in whole muscle lysates, their expression remained stable within the remaining mitochondria. These findings suggest that, although fewer mitochondria are present, those that remain retain functional integrity. Notably, this preservation of mitochondrial quality is paralleled by the maintenance of type I (oxidative) and type II (glycolytic) muscle fibers between active and hibernating states, indicating that muscle structure and metabolic properties are maintained during hibernation. Supporting this, ATP levels were found to be elevated in hibernating muscle (personal data), despite reductions in ATP turnover and ATPase activity at the myosin level observed in the same brown bear population [30]. Similar reductions in ATP utilization have been observed in small hibernators (garden dormouse, *Eliomys quercinus*, and 13‐lined ground squirrel, *Ictidomys tridecemlineatus* ) [31], where myosin ATP consumption is markedly suppressed during torpor. Together, these findings indicate that ATP availability in the muscle is unlikely to limit muscle function during hibernation; rather, conserved ATP turnover may lessen the demand for ATP production.

Collectively, these data highlight how mitochondrial energetics are maintained in a dormant but responsive state. The skeletal muscle of hibernating brown bears contains fewer mitochondria, but those that remain are equipped to rapidly resume energy production in case of an arousal. In small hibernators, mitochondrial regulation during torpor is dynamic and tissue‐specific. In species such as thirteen‐lined ground squirrels, respiratory rates have been measured across multiple tissues—muscle, liver, heart, brain, and brown adipose—revealing both strong suppression (especially in liver) and rapid reactivation during periodic arousals [12, 32, 33, 34]. This was accompanied by tissue‐dependent changes in mitochondrial density, with either an increase (e.g., Daurian ground squirrels, *Spermophilus dauricus* ) or unchanged (e.g., thirteen‐lined ground squirrels) abundance during hibernation [35, 36]. Differences in the molecular equipment of mitochondria have also been reported to be tissue‐specific in small hibernating rodents [32]. Because our study focused solely on skeletal muscle, such multi‐tissue responses cannot be directly compared; nevertheless, our results suggest that bears follow a distinct strategy, with reduced mitochondrial density but preserved proteomic integrity and a maintained capacity for rapid reactivation despite continuous torpor. For more details about the mechanisms of mitochondrial metabolic suppression during hibernation in ground squirrels, the readers should refer to the review article of Staples et al. [34].

Notably, prior work in this bear population has shown seasonal downregulation of the endocannabinoid system in both muscle and adipose tissue [37], which may promote energy mobilization, support torpor maintenance, and enhance responsiveness to environmental stimuli. Although the precise prevalence of emergency arousals in wild brown bears remains unclear, data in captive and wild animals suggest a notable sensitivity to winter disturbances [38]. For example, a study in Sweden reported a 22% rate of den abandonment in a single season, likely because of human activities [39], indicating that emergency arousal may be common.

These energetic changes contrast with a 70% suppression of hepatic mitochondrial respiration in small mammalian hibernators, arising from inhibition of ETC complexes I and II activities [40], without changes in mitochondrial content [41]. In skeletal muscle of thirteen‐lined ground squirrels, mitochondrial respiration decreases moderately during torpor (~30%) under specific substrate and temperature conditions, despite no apparent reduction in mitochondrial content [12]; in arctic ground squirrels, skeletal muscle mitochondrial respiration appears unchanged during hibernation [13]. Some studies link this depressed mitochondrial respiration to hydrogen sulfide (H_2_S) metabolism and the inhibition of cytochrome *c* oxidase [42]. Interestingly, in our bear data, we did not observe such a marked decrease in OXPHOS capacity, despite an upregulation of sulfur metabolism pathways in the whole‐muscle proteome. This may point to compensatory mechanisms unique to bears, which maintain continuous torpor without periodic arousals, unlike small hibernators who undergo frequent metabolic reactivations driven by arousal.

### 4.2 Skeletal Muscle Mitochondria Maintain the Ability to Use Both Carbohydrate and Fatty Acid Substrates That Enter the TCA Cycle During Hibernation

Despite systemic suppression of carbohydrate oxidation during hibernation, the mitochondrial capacity to oxidize carbohydrate substrates ex vivo was preserved (no temperature effect) and even slightly enhanced at 33°C, which corresponds to the mean body temperature in hibernating bears [43]. This preservation aligns with previous findings in this Swedish bear population, which showed maintenance of glycolytic machinery in winter muscle, likely supported by liver gluconeogenesis and muscle glycogen mobilization [15]. Together, these processes may allow for intermittent glucose use even in a hypometabolic state, subsequently feeding the Cori cycle as previously suggested [15].

This observation suggests that the suppression of carbohydrates oxidation per mitochondria predominantly occurs post‐transcriptionally rather than by reductions in enzyme abundance. A potential candidate known for its rapid responsiveness is PDK4, which, through phosphorylation and inhibition of pyruvate dehydrogenase complex (PDC), limits the entry of glycolytic intermediates into the Krebs cycle. The previously reported presence of glycogen stores in winter muscle further supports the notion that this mechanism enables bears to rapidly switch between fatty acid oxidation and glucose utilization depending on immediate energetic demands [15].

### 4.3 Increased Reliance on Complex II: A Temperature‐Sensitive Mechanism to Preserve Mitochondrial Energetics During Hibernation

Our findings reveal a shift in mitochondrial respiration favoring Complex II in the ETC during hibernation. SDH activity remained unchanged in winter muscle despite lower protein expression in the skeletal muscle lysate, and no change was observed in SDH protein expression in isolated mitochondria, suggesting that post‐translational modifications or enhanced functional efficiency maintain Complex II activity. Notably, 6 out of 8 animals exhibited increased SDH activity during hibernation, suggesting that skeletal muscle retains a capacity for rapid energy production.

In small hibernators such as thirteen‐lined ground squirrels [28] and Arctic ground squirrels [44], succinate oxidation is actively inhibited during torpor, particularly in liver and skeletal muscle, presumably to limit reactive oxygen species (ROS) production. Importantly, this inhibition appears strongly temperature‐dependent: SDH activity is suppressed when assayed at 37°C but not at 10°C [28]. In contrast, our SDH assays were performed at 25°C, and we observed no evidence of SDH inhibition in bears. These differences suggest that SDH inhibition may contribute to initiating mitochondrial suppression in small hibernators, whereas in bears, who do not undergo deep torpor–arousal cycles, SDH does not appear necessary to maintain metabolic suppression. The hypothesis proposed for torpid ground squirrels, that is, mitochondria maintain oxidative capacity above a minimal functional threshold despite low succinate levels [28], may therefore also apply to bears, whose mitochondria remain competent even under reduced metabolic demand.

The functional relevance of preserved Complex II flux in winter bears may be explained by anaplerotic pathways capable of sustaining succinate supply despite reduced TCA cycle activity [45]. Amino acids such as valine, isoleucine, methionine and threonine can be catabolized into propionyl‐CoA and then succinyl‐CoA, ultimately generating succinate. Glutamate can also be converted into α‐ketoglutarate and subsequently into succinyl‐CoA. Our mitochondrial proteomic data support this possibility: several enzymes involved in these pathways—including branched‐chain amino‐acid aminotransferase, short/branched‐chain acyl‐CoA dehydrogenase, aspartate aminotransferase, 3‐mercaptopyruvate sulfurtransferase, propionyl‐CoA carboxylase α/β, and succinate‐CoA ligase—are maintained in winter mitochondria (Table S3). This provides a plausible mechanism for sustaining Complex II activity even under reduced TCA flux.

Another observation to provide commentary concerns SDH and its function. It should be stated that SDH function following hibernation may be preserved because it is fully encoded by nuclear DNA and is not affected by the sharp drop in mtDNA content observed during hibernation. In contrast, complexes containing mitochondrial‐encoded subunits (Complexes I, III, and IV) appear more vulnerable to mtDNA decline, contributing to the overall decrease in OXPHOS capacity [3]. Whether all mitochondria retain similar mtDNA content or whether distinct mitochondrial subpopulations (e.g., subsarcolemmal vs. intermyofibrillar mitochondria) are differentially affected remains an open question. It is conceivable that some mitochondria become “silent” or dormant, with diminished mtDNA content and reduced functional complexes (except for Complex II), allowing a rapid reactivation upon arousal without requiring full mitochondrial biogenesis. Alternatively, a true mitochondrial loss could occur. Future ultrastructural and functional analyses are needed to explore these possibilities.

In addition to these potential structural changes, the decrease in proton leak observed in our study suggests a reduction in the activity of mitochondrial transporters, such as malate/fumarate transport and possibly carnitine palmitoyltransferases (CPT1 and CPT2), which regulate fatty acid entry into mitochondria. This aligns with the observed trend toward downregulation of SLC25A11 and SLC25A13 transporters (involved in the malate–aspartate shuttle), both here and previously reported in hibernating brown bear muscle lysates [15]. Interestingly, the expression of SLC25A11 and SLC25A13 remained unchanged in isolated mitochondrial fractions, as determined by our proteomic analysis, suggesting potential regulation at the level of transporter activity or localization rather than protein abundance.

### 4.4 Limited but Targeted Modulation of the Mitochondrial Proteome During Hibernation to Sustain Complex II Activity

Our findings collectively support the hypothesis that mitochondrial respiration during hibernation shifts toward a more FADH_2_‐driven system, prioritizing Complex II while conserving energy [4]. Complex II may also partially compensate for reduced Complex I activity, as increased electron flow through Complex II has been reported in Complex I‐deficient mice [46, 47].

In human obesity and type 2 diabetes, metabolically unhealthy individuals with overweight or obesity exhibit increased circulating succinate levels [48, 49], which are linked to impaired carbohydrate metabolism and mitochondrial dysfunction [50]. Succinate, traditionally viewed as a mitochondrial metabolite, also acts as a signaling molecule that influences metabolic pathways and oxidative stress. Its accumulation is associated with insulin resistance and inflammatory responses, contributing to the metabolic complications observed in metabolic diseases [50]. Interestingly, these metabolic shifts in humans are offset by an increased expression of Complex II, which helps sustain ATP production despite impaired carbohydrate metabolism. This suggests that mitochondrial response in bears during prolonged inactivity may resemble metabolic responses in human with obesity, insulin resistance and/or sedentary lifestyles.

Several mechanisms appear to support Complex II activity during hibernation. FADH_2_, its key electron donor, is produced both through fatty acid oxidation—predominant during hibernation –and the glycerol‐phosphate shuttle; the latter of which also regulates NADH availability. Although the two enzymes of this shuttle, including mitochondrial and cytosolic glycerol‐3‐phosphate dehydrogenase (GPDH), showed no seasonal variation in our proteomic data generated from both the isolated mitochondria and whole muscle, their presence may help sustain FADH_2_ supply. The maintained expression of the dicarboxylate carrier SLC25A10 in mitochondria could similarly support succinate entry into the mitochondria, ensuring continued electron flow through Complex II under hypometabolic conditions. Together, these responses may help preserve mitochondrial ATP production while minimizing energy cost.

Additional regulation may involve oxaloacetate tautomerase FAHD1, which converts enol‐oxaloacetate, a strong inhibitor of SDH [51], into its keto form of oxaloacetate, thereby maximizing aerobic respiration efficiency [52]. Its significantly increased abundance in the mitochondrial fraction of hibernating bear muscle likely contributes to maintaining muscle SDH activity. Oxaloacetate abundance has been found to remain stable in the liver of thirteen‐lined ground squirrels ( *I. tridecemlineatus* ) during hibernation [53]. However, measuring total oxaloacetate abundance may not fully capture its functional role. As such, future studies should determine its relative abundance and the activity of its different tautomers in various tissues of hibernators.

Although lactate can enter mitochondria independently of the mitochondrial pyruvate carrier [54] and has been reported to be an effective fuel for stimulating muscle mitochondrial coupling efficiency, our data do not support this role during hibernation. Indeed, lactate oxidation was shown to depend on the presence of lactate dehydrogenase (LDH) in mitochondria [55]. Despite unchanged LDH isoform A expression in whole muscle, which is in agreement with previous results [15], its significant decrease in the mitochondrial fraction in winter suggests that lactate is not oxidized in bear muscle mitochondria during torpor. That said, we cannot rule out the possibility that some of the lactate produced during hibernation [15] is diverted to the mitochondria where it might stimulate the activity of the mitochondrial ETC independently of LDH, an ability that has been reported to involve the activation of pyruvate dehydrogenase (PDH), then an increase of pyruvate entry and oxidation into the mitochondrial TCA cycle [54], potentially counteracting PDK4‐mediated inhibition. This suggests a possible regulatory balance between lactate signaling and PDH inhibition, which merits further study. Future research should quantify mitochondrial lactate concentration and test this hypothesis in hibernators.

Muscle atrophy has been linked to mitochondrial dysfunction, notably through inhibition of key mitochondrial fusion‐fission regulators such as the inner mitochondrial membrane fusion protein Optic Atrophy 1 (OPA1) and the fission protein Dynamin Related Protein 1 (DRP1, or DNM1L) in mice [56, 57]. In hibernating bears, OPA1 expression remained unchanged in both the whole muscle lysate and the mitochondrial fraction. MFN2, an outer mitochondrial membrane fusion protein, was only detected in the mitochondrial fractions, with no seasonal variations. Although DNM1L was threefold lower in whole muscle lysates during hibernation, it was undetectable in mitochondrial fractions, suggesting minimal recruitment of DRP1/DNM1L to the mitochondrial membrane. Among other fission/fusion proteins, Fission protein 1 (FIS1) showed a non‐significant decrease in muscle lysates but not in isolated mitochondria, whereas Mitochondrial Fission Process 1 (MTFP1), which is involved in mitochondrial division, was markedly reduced in isolated mitochondria only. This downregulation of MTFP1 may help preserve mitochondrial energetics during hibernation. Indeed, while *Mtfp1* deletion causes cardiomyopathy in mice [58], hepatocyte‐specific deletion enhances respiration without adverse effects [59] and protects against liver apoptosis by inhibiting mitochondrial permeability transition pore (mPTP) opening [59]. Thus, the selective reduction of MTFP1 in bear muscle mitochondria may support mitochondrial integrity and muscle mass preservation in winter. Future work should investigate whether mPTP is affected in the bear muscle during hibernation and contributes to this protective mechanism.

### 4.5 Limitations & Future Studies

A few limitations should be acknowledged. First, respiratory rates are temperature‐dependent, and the mismatch in assay temperatures between isolated mitochondria (25°C) and permeabilized fibers (33°C–37°C) limits direct quantitative comparison. In addition, previous work in small hibernators has shown that isolated mitochondria and permeabilized fibers can yield different results [60], emphasizing the need for cautious interpretation. The choice of 25°C for isolated mitochondria was guided by both methodological consistency with prior studies and strict logistical constraints in the field. Because of low mitochondrial yield, especially in winter, we place greater confidence in the permeabilized fiber data, although the seasonal comparison within the isolated‐mitochondria dataset remains valid. Future studies should assess mitochondrial respiration at a minimum of three experimental temperatures to enable proper calculation of Q_10_ eand to better characterize the temperature sensitivity of mitochondrial energetics. Second, we did not directly measure NADH or FADH_2_ concentrations or assess the enzymatic activities and post‐translational modifications of key regulatory nodes such as the pyruvate dehydrogenase complex (PDC) and related metabolic enzymes. Such analyses would help clarify the mechanisms driving substrate preference during hibernation. Third, we did not quantify muscle or mitochondrial lactate concentrations, nor did we measure the expression of enol‐oxaloacetate (enol‐OAA), a potent inhibitor of SDH. Future targeted or untargeted metabolomics analyses of skeletal muscle would provide deeper insights into metabolic regulation during hibernation. Fourth, to better understand the temporal dynamics of mitochondrial regulation, it will be important to measure mitochondrial respiration, substrate oxidation capacity, and associated molecular markers during the early entrance into hibernation and throughout the first weeks of dormancy. Such longitudinal studies could reveal whether mitochondrial suppression is actively initiated at hibernation onset and whether its reversal during natural arousal follows a gradual or rapid trajectory, distinct from emergency‐induced arousals.

## 5 Conclusions

Hibernating brown bears maintain skeletal muscle mitochondrial energetics during torpor through a regulated, multifaceted strategy. Despite reduced mitochondrial density, core respiratory machinery remains intact, with increased reliance on Complex II and preserved SDH activity, representing a temperature‐sensitive maintenance of electron flow while limiting ATP production. Proteomic analyses revealed maintained mitochondrial components and selective regulation of transporters, potentially protecting against skeletal muscle atrophy while retaining the ability to oxidize fatty acids and carbohydrates, especially at lower temperatures. These responses keep skeletal muscle energy‐efficient and quiescent during hibernation yet primed for rapid reactivation, offering insights into preserving mitochondrial energetics during prolonged inactivity.

## Author Contributions

A.B., E.L., F.B. acquired funding; A.B., D.O., E.L. and F.B. conceptualized the study, A.B., D.O., E.L. and F.B. developed the experimental protocols; A.B., J.N., C.B., L.C., A.G., C.C.‐G., I.C., A.L.E., J.M.A., J.K., D.O., E.L., and F.B. acquired samples and data; A.B. and F.B. ran the statistical analyses; A.B., J.N., D.O., E.L. and F.B. analyzed the data; A.B. and F.B. prepared the figures; A.B., J.N., D.O., E.L. and F.B. drafted the paper; all authors proofread the final version of the manuscript.

## Funding

The long‐term funding of SBBRP has come primarily from the Swedish Environmental Protection Agency and the Norwegian Environment Agency. This research was funded by the French National Space Agency (CNES, BEAR2MAN project), the French National Research Agency (ANR; B‐STRONG project), the University of Strasbourg (H2E project), and the French National Center for Scientific Research (Centre National de la Recherche Scientifique ‐ CNRS) Mission des Initiatives Transversales et Interdisciplinaires (MITI).

## Ethics Statement

The study was approved by the Swedish Ethical Committee on Animal Experiments, Uppsala, Sweden (applications Dnr C3/2016 and Dnr C18/2015), the Swedish Environmental Protection Agency (NV‐0741‐18), and the Swedish Board of Agriculture (Dnr 5.2.18‐3060/17). All procedures complied with Swedish laws and regulations.

## Conflicts of Interest

The authors declare no conflicts of interest.

## Supporting information


**Data S1:** Supporting Information.

**Figure S1:** Mitochondrial respiration of mitochondria isolated from skeletal muscle biopsy collected in active and hibernating brown bears. Isolated mitochondrial respiration normalized for protein content in the presence of either carbohydrate (A; CHO SUIT) or fatty acid (C; FAT SUIT) supported substrates in skeletal muscle collected from summer physically active (*n* = 8, summer) and winter hibernating bears (*n* = 8, winter) during experiments run at 25°C. Ratios between coupled (OXPHOS (CI)) and uncoupled (LEAK) respiration, between maximal respiration through Complex I and Complex II (ETS (CI + CII)) and coupled respiration (OXPHOS (CI)), and between maximal respiration through Complex I and Complex II (ETS (CI + CII)) and respiratory capacity through Complex II only when Complex I is inhibited (ETS (CII)) in presence of either carbohydrates (C) or fatty acid (D) are also presented. ETS (CI + CII) maximal respiration through Complex I and Complex II; ETS (CII), respiratory capacity through Complex II only when Complex I is inhibited; OXPHOS (CI), ADP‐coupled respiration through Complex I; OXPHOS (CI + CII), ADP‐coupled respiration through Complex I and Complex II. Differences between summer and winter were statistically analyzed using paired *T*‐test. Data are expressed as the mean ± SEM, Red bars, summer; blue bars, winter. Individual response represented by dots (summer) and diamonds (winter).

**Table S1:** Bear characteristics.

**Table S2:** List of 1335 proteins quantified using label‐free proteomics in skeletal muscle from hibernating vs. summer‐active brown bears ( *Ursus arctos* ).

**Table S3:** List of 720 proteins quantified using label‐free proteomics in muscle mitochondrial extracts from hibernating vs. summer‐active brown bears ( *Ursus arctos* ).

**Table S4:** Permeabilized muscle fibers respiratory rates measured in active and hibernating bears at 33°C and 37°C.

**Table S5:** Skeletal muscle isolated mitochondria respiratory rates measured in active and hibernating bears at 25°C.

## Data Availability

The data that support the findings of this study are available on request from the corresponding author. The data are not publicly available due to privacy or ethical restrictions.
