# Supplementary material for: The Preservation of Muscle Mitochondrial Machinery During Hypometabolic Hibernation in Scandinavian Brown Bears ( Ursus arctos )
Source: Acta Physiol (Oxf). 2026 Feb 23;242(4):e70177. doi: 10.1111/apha.70177 (PMC12926787; doi:10.1111/apha.70177)
Supplement: Supplementary file 1 — Data S1: Supporting Information. [file APHA-242-e70177-s006.docx]

**Supplementary materials & methods**

**Proteomics of bear muscle and mitochondrial enriched fractions**

Unless otherwise specified, all chemicals were purchased from Sigma Aldrich (St. Louis, MO, USA).

***Preparation of bear muscle protein extracts.*** Frozen bear muscle samples (n=7 per group) were ground under liquid nitrogen (30 sec., 30 Hz) using a laboratory ball mill (MM400, Retsch, Eragny sur Oise, France). The powders obtained were placed in lysis buffer (6M Urea, 0.1 M ammonium bicarbonate, 2% CHAPS, protease inhibitors 1.6 µM to 2 mM, 25 mM Tris). After incubation during 45 minutes at 37 °C, the samples were centrifuged to remove cell debris. Six volumes of cold acetone were then added to the supernatants that were kept at -20°C overnight. Precipitated proteins were pelleted by centrifugation (20 minutes, 4°C, 13500 x g), washed one time with acetone and then resuspended in sample buffer (8M urea, 0.1 M ammonium bicarbonate).

***Preparation of bear muscle mitochondrial protein extracts.*** Frozen mitochondrial fractions (N=4 per group) were resuspended in a lysis buffer (urea 8M, thiourea 2M, dithiothreitol [DTT] 1%, protease inhibitors 1.6 µM to 2 mM, 50 mM Tris pH 7.4). After incubation during 45 min at room temperature, eight volumes of cold acetone were added to samples that were kept at -20°C overnight. Precipitated proteins were pelleted by centrifugation (20 minutes, 4°C, 13500 x g), washed one time with acetone and then dissolved in Laemmli buffer (10 mM Tris pH 6.8, 1 mM EDTA, 5% β- mercaptoethanol, 5% SDS, 10% glycerol).

***Protein electrophoresis and digestion.*** Total protein concentration was determined using the RC DC Protein Assay kit from Bio-Rad (Hercules, CA, USA). At this stage, a reference sample comprising equal amounts of all protein extracts was made (one reference sample for muscle protein extracts and another for muscle mitochondrial protein extracts), to be analyzed regularly during the whole experiment and allow QC-related measurements. Total muscle protein extracts and muscle mitochondrial protein extracts were analysed in two separate experiments.

Total muscle proteins on one hand (fifty µg) and mitochondrial proteins on another hand (six µg) were first loaded onto SDS-PAGE stacking gels (4% polyacrylamide) and electrophoresed for 20 minutes at 35 V. After protein fixation (50% ethanol, 3% phosphoric acid) during 15 min, staining using colloidal Coomassie Blue allowed visualization of a stacked protein band. The stacked protein band was excised from the gel, as well as the part of the gel above this band. Destaining was performed using 75% acetonitrile/25% 25 mM ammonium hydrogen carbonate 25 mM (four times 5 minutes at RT), and dehydration using pure acetonitrile (5 minutes at RT). Proteins were then reduced and alkylated in-gel using 10 mM DTT in 25 mM ammonium hydrogen carbonate (30 minutes at 60°C then 30 minutes at RT) and 55 mM iodoacetamide in 25 mM ammonium hydrogen carbonate (20 minutes at RT in the dark), respectively. Gel pieces were washed using 25 mM ammonium hydrogen carbonate (5 minutes at RT) then acetonitrile (5 minutes at RT), and these steps were repeated several times. Finally, dehydration used acetonitrile (2 x 5 minutes at RT), and in-gel digestion of proteins was performed overnight at 37°C using trypsin (Promega, Madison, WI, USA; 1 µg per band for total proteins and 60 ng per band for mitochondrial proteins). After trypsin digestion, the resulting peptides were extracted twice at 450 rpm on an orbital shaker during 45 min using 60% acetonitrile/0.5% formic acid in water, then during 15 min using 100% acetonitrile. At this stage, a set of reference peptides (iRT kit; Biognosys AG, Schlieren, Switzerland) was added to mitochondrial peptide extracts (3µL/sample after resuspension in 500 mL of 20% acetonitrile/1% formic acid) for QC-related measurements. The volume of mitochondrial peptide extracts was first reduced using a vacuum centrifuge (SpeedVac, Savant, Thermoscientific, Waltham, MA, USA) then adjusted to 20 μL using 1% acetonitrile/0.1% formic acid in water. For total proteins, vacuum-drying was performed (SpeedVac, Savant, ThermoFisher Scientific) and tryptic peptides were suspended in 100 µL of 0.1% formic acid in water. An in-house digest of the HELA cell line was used for QC-related measurements.

***NanoLC-MS/MS analysis.*** Samples were analysed on a nanoUPLC-system (nanoAcquity, Waters, Milford, MA, USA) coupled to a quadrupole-Orbitrap hybrid mass spectrometer (Q-Exactive HF-X for total proteins and Q-Exactive plus for mitochondrial proteins, Thermo Scientific, San Jose, CA). The system was fully controlled by XCalibur software (v4.0.27.19 and v3.0.63; Thermo Fisher Scientific). Concentration/desalting was first performed by loading of 3µL of sample (total proteins) or 2 μL of sample (mitochondrial proteins) on a nanoEase M/Z Symmetry precolumn (C18, 100 Å, 5 μm, 180 μm × 20 mm; Waters) using 0.1% formic acid in water (solvent A, 99%) and 0.1% formic acid in acetonitrile (solvent B, 1%) at a flow rate of 5 μl/min for 3 min. Peptide elution was then performed at a flow rate of 400 nL/min using a nanoEase M/Z BEH column (C18, 130 Å, 1.7 μm, 75 μm x 250 mm; Waters) maintained at 60 °C and a solvent gradient from 1 to 40% of B in 150 minutes (total protein samples) or from 1 to 8% of B in 2.5 minutes then 8 to 40% of B in 75.5 minutes (mitochondrial samples). Injection of samples from winter and summer bears was performed alternately, but in a random fashion, and the reference sample was analysed five (total protein experiment) or three (mitochondrial experiment) times throughout the experiment. For the total protein experiment, the digest of HELA cells was injected 6 times throughout the experiment. To reduce carry-over, the column was washed with 90% acetonitrile during 6 minutes then one or two solvent blanks were run in between each sample. Peak intensities and retention times of reference peptides were monitored in a daily fashion.

The Q-Exactive Plus and HF-X were operated in positive ion mode with source temperature set to 250 °C and spray voltage to 2.2 kV (HF-X) and 2.0 kV (Plus). With the Q-Exactive HF-X and Plus, full-scan MS spectra (350-1500 m/z and 300−1800 m/z, respectively) were acquired at a resolution at m/z 200 of 120,000 and 70,000, respectively. A maximum injection time of 50 ms, and an AGC target value of 3 × 10^6^ ions with the lock-mass option being enabled (445.12002 m/z). Up to 20 (HF-X) or 10 (Plus) most intense precursors (number of charges > 1) per full scan were isolated using a 2 m/ z window and fragmented using higher energy collisional dissociation (HCD, normalized collision energy of 27), and dynamic exclusion of already fragmented precursors was set to 60 s. MS/MS spectra were acquired with a resolution of 15,000 (HF-X) and 17,500 (Plus) at m/z 200, a maximum injection time of 60 ms (HF-X) or 100 ms (Plus), and an AGC target value of 1 × 10^5^.

***NanoLC-MS/MS data processing.*** MS raw data were processed using MaxQuant (v1.6.11.0 or v2.6.3.0) ^1^. Peak lists were created using default parameters and searched using Andromeda search engine implemented in MaxQuant against a protein database created using the MSDA software suite ^2^. The database contained RefSeq sequences for *Ursus arctos* proteins (GCF_003584765.1 and GCF_023065955.2 assemblies). Within Andromeda search engine, sequences of decoy (reverted) sequences for all all *Ursus arctos* proteins and of common contaminants like keratins and trypsin (247 entries) were added to the database. For the first search, precursor mass tolerance was set to 20 ppm and it was set to 4.5 ppm for the main search after recalibration. Fragment ion mass tolerance was set to 20 ppm. Carbamidomethylation of cysteine residues was considered as fixed and oxidation of methionine residues and acetylation of protein N-termini as variable modifications during the search. A maximum number of one missed cleavage was tolerated, and a false discovery rate (FDR) of 1% for both peptide spectrum matches (minimum length of seven amino acids) and proteins was accepted during identification. The proteins identified with a single peptide or with a negative score were discarded from identification data, as well as decoy hits and potential contaminants.

For quantitative purposes, data normalization and protein abundance estimation was performed using the MaxLFQ (label-free quantification) option implemented in MaxQuant ^1^, using a “minimal ratio count” of one. The “Match between runs” option was enabled using a 0.7-min time window after retention time alignment. Both unmodified and modified (acetylation of protein N-termini and oxidation of methionine residues) peptides were considered for quantification, while shared peptides were excluded. All other MaxQuant parameters were set as default. Proteins that were retained for further analysis were those for which quantitative values were obtained from a minimum of one (total protein experiment, only 29 proteins quantified with a single peptide) or two (mitochondrial experiment) unique peptides, and exhibiting no more than three (total protein experiment) or only one (mitochondrial experiment) missing value per group. Proteins absent in a given group (i.e. not detected at all) were also retained. The mass spectrometry proteomics data have been deposited to the ProteomeXchange Consortium via the PRIDE ^3^ partner repository with the dataset identifiers PXD060503 for the total protein experiment and PXD036916 for the mitochondrial experiment.

***Proteomic data functional analysis.*** To identify functionally relevant biological processes differently regulated in skeletal muscle of bears during summer and winter, enrichment and functional annotation analyses of differential proteomics data were performed using the interactome analysis and CAME (ID Conversion, Annotation, Membership, function Enrichment) workflow for the annotation enrichment analysis applied in Metascape (https://metascape.org/; accessed on 21 January 2025). Default parameters were used. Using mainly the Biogrid database, information about physical protein-protein interaction was first extracted and a protein interaction network was built. A molecular complex detection (MCODE) algorithm was then applied to this network to identify neighborhoods where proteins were densely connected. Ontology enrichment analysis, which was performed using the most popular annotation sources, e.g., GO and Kegg, but alsoWikiPathways, Reactome, Corum and a series of other databases, was finally applied to each MCODE neighborhoods to assign them a biological function on the basis of the top-three functional enriched terms, when available. Networks were visualized using Cytoscape software v3.8.2.^4^.

***QC-related measurements.***

QC-related measurements indicated stable performances of the analysis systems all along the experiments, with very low mean coefficients of variation (CVs) for retention times of 5 major ions from HELA cell analysis (0.5%, total protein experiment) and iRT peptides over all injections (2.6%, mitochondrial experiment). For all LFQ values determined in the repeatedly injected reference samples, a mean CV of 17.4% and 18% was obtained for the total protein experiment and the mitochondrial experiment, respectively.

1 Cox, J. *et al.* Accurate proteome-wide label-free quantification by delayed normalization and maximal peptide ratio extraction, termed MaxLFQ. *Mol Cell Proteomics* **13**, 2513-2526 (2014). <https://doi.org/10.1074/mcp.M113.031591>

2 Carapito, C. *et al.* MSDA, a proteomics software suite for in-depth Mass Spectrometry Data Analysis using grid computing. *Proteomics* **14**, 1014-1019 (2014). <https://doi.org/10.1002/pmic.201300415>

3 Vizcaino, J. A. *et al.* 2016 update of the PRIDE database and its related tools. *Nucleic Acids Res* **44**, D447-456 (2016). <https://doi.org/10.1093/nar/gkv1145>

4 Shannon, P. *et al.* Cytoscape: a software environment for integrated models of biomolecular interaction networks. *Genome Res.* **13**, 2498-2504 (2003). <https://doi.org/10.1101/gr.1239303>
