# Supplementary figures and images for: The Preservation of Muscle Mitochondrial Machinery During Hypometabolic Hibernation in Scandinavian Brown Bears ( Ursus arctos )
Source: Acta Physiol (Oxf). 2026 Feb 23;242(4):e70177. doi: 10.1111/apha.70177 (PMC12926787; doi:10.1111/apha.70177)

**A**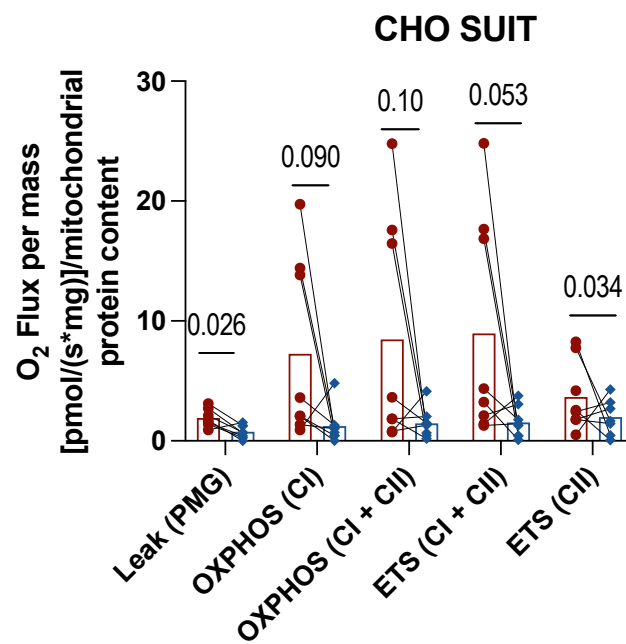**B**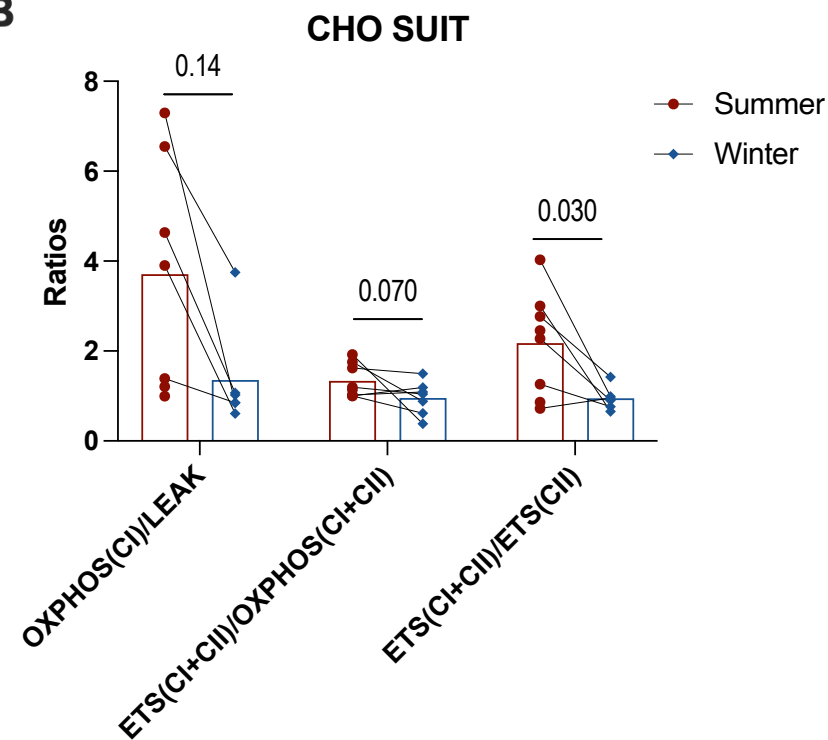**C**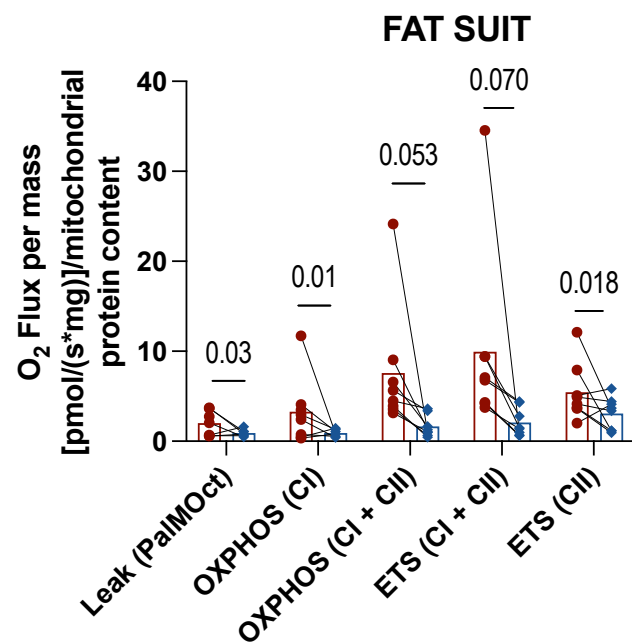**D**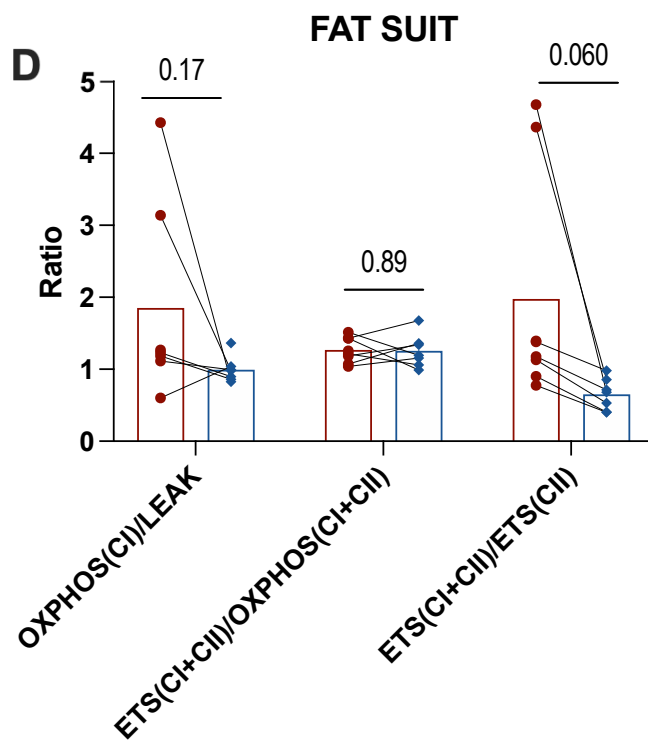

Supplement: Supplementary file 2 — Figure S1: Mitochondrial respiration of mitochondria isolated from skeletal muscle biopsy collected in active and hibernating brown bears. Isolated mitochondrial respiration normalized for protein content in the presence of either carbohydrate (A; CHO SUIT) or fatty acid (C; FAT SUIT) supported substrates in skeletal muscle collected from summer physically active (n = 8, summer) and winter hibernating bears (n = 8, winter) during experiments run at 25°C. Ratios between coupled (OXPHOS (CI)) and uncoupled (LEAK) respiration, between maximal respiration through Complex I and Complex II (ETS (CI + CII)) and coupled respiration (OXPHOS (CI)), and between maximal respiration through Complex I and Complex II (ETS (CI + CII)) and respiratory capacity through Complex II only when Complex I is inhibited (ETS (CII)) in presence of either carbohydrates (C) or fatty acid (D) are also presented. ETS (CI + CII) maximal respiration through Complex I and Complex II; ETS (CII), respiratory capacity through Complex II only when Complex I is inhibited; OXPHOS (CI), ADP‐coupled respiration through Complex I; OXPHOS (CI + CII), ADP‐coupled respiration through Complex I and Complex II. Differences between summer and winter were statistically analyzed using paired T‐test. Data are expressed as the mean ± SEM, Red bars, summer; blue bars, winter. Individual response represented by dots (summer) and diamonds (winter). [file APHA-242-e70177-s007.pdf]
