# Supplementary material for: The Preservation of Muscle Mitochondrial Machinery During Hypometabolic Hibernation in Scandinavian Brown Bears ( Ursus arctos )
Source: Acta Physiol (Oxf). 2026 Feb 23;242(4):e70177. doi: 10.1111/apha.70177 (PMC12926787; doi:10.1111/apha.70177)
Supplement: Supplementary file 3 — Table S1: Bear characteristics. [file APHA-242-e70177-s003.docx]

**Supplemental Table 1. Bear characteristics**

| **Bear Id** | **Sex** | **Age (y)** | **Body mass in summer (kg)** | **Body mass in Winter (kg)** |
| --- | --- | --- | --- | --- |
| W1814 | M | 2 | 43.3 | 54.0 |
| W1812 | M | 2 | 49.8 | 42.0 |
| W1813 | F | 2 | 39.0 | 33.0 |
| W1803 | F | 2 | 38.8 | 33.0 |
| W1802 | M | 2 | 47.5 | 39.0 |
| W1806 | F | 2 | 32.0 | 29.5 |
| W1707 | F | 3 | 63.6 | 61.0 |
| W1709 | F | 3 | 56.4 | 54.0 |

F, female ; M, male
