# Supplementary material for: The Preservation of Muscle Mitochondrial Machinery During Hypometabolic Hibernation in Scandinavian Brown Bears ( Ursus arctos )
Source: Acta Physiol (Oxf). 2026 Feb 23;242(4):e70177. doi: 10.1111/apha.70177 (PMC12926787; doi:10.1111/apha.70177)
Supplement: Supplementary file 6 — Table S4: Permeabilized muscle fibers respiratory rates measured in active and hibernating bears at 33°C and 37°C. [file APHA-242-e70177-s002.docx]

**Supplemental Table 4. Permeabilized muscle fibers respiratory rates measured in active and hibernating bears at 33°C and 37°C**

|  | **Summer physically active bears (n=8)** | | **Winter hibernating**  **bears (n=8)** | | **Season effect**  **(p-value)** | **Temperature effect**  **(p-value)** | **Season*Temperature effect**  **(p-value)** |
| --- | --- | --- | --- | --- | --- | --- | --- |
|  | **33°C** | **37°C** | **33°C** | **37°C** |  |  |  |
| **Citrate synthase expression (A.U.)** |  | 1.39 (1.01) |  | 1.17 (1.01) | 0.027 |  |  |
| **CHO SUIT- O2 flux per mass uncorrected for citrate synthase abundance expressed in pmol/(s*mg)** | | | | | | | |
| **Leak (PMG)** | 19.73 (1.31) | 29.38 (1.31) | 8.85 (1.31) | 7.62 (1.31) | 0.0005 | 0.614 | 0.291 |
| **OXPHOS (CI)** | 59,10 (1.38) | 79,96 (1.38) | 12.21 (1.38) | 8.43 (1.38) | <0.001 | 0.800 | 0.245 |
| **OXPHOS (CI+CII)** | 68.37 (1.32) | 91.22 (1.32) | 25.29 (1.32) | 21.01 (1.32) | 0.0016 | 0.760 | 0.291 |
| **ETS (CI+CII)** | 63,84 (1.31) | 80.71 (1.31) | 23.78 (1.31) | 20.14 (1.31) | 0.0008 | 0.838 | 0.350 |
| **ETS (CII)** | 24.24 (1.23) | 39.77 (1.23) | 17.44 (1.23) | 13.71 (1.23) | 0.006 | 0.431 | 0.061 |
| **ROX** | 7.93 (1.35) | 10.46 (1.35) | 5.13 (1.35) | 6.25 (1.35) | 0.101 | 0.445 | 0.894 |
| **CHO SUIT- O2 flux per mass corrected for citrate synthase abundance expressed in pmol/(s*mg)/CS expression** | | | | | | | |
| **Leak (PMG)** | 71.32 (1.37) | 106.23 (1.37) | 62.58 (1.37) | 53.91 (1.37) | 0.121 | 0.542 | 0.323 |
| **OXPHOS (CI)** | 213.71 (1.45) | 278.30 (1.45) | 86.41 (1.45) | 59.62 (1.45) | 0.001 | 0.766 | 0.309 |
| **OXPHOS (CI+CII)** | 247.25 (1.36) | 329.84 (1.36) | 178.90 (1.36) | 148 (1.36) | 0.083 | 0.755 | 0.270 |
| **ETS (CI+CII)** | 230.83 (1.37) | 291.84 (1.37) | 168.28 (1.37) | 142.47 (1.37) | 0.101 | 0.829 | 0.331 |
| **ETS (CII)** | 87.65 (1.30) | 143.81 (1.30) | 123.37 (1.30) | 97.02 (1.30) | 0.901 | 0.382 | 0.039 |
| **ROX** | 28.67 (1.42) | 37.82 (1.42) | 36.27 (1.42) | 44.24 (1.42) | 0.531 | 0.457 | 0.901 |
| **FAT SUIT- O2 flux per mass uncorrected for citrate synthase abundance expressed in pmol/(s*mg)** | | | | | | | |
| **Leak (PMOct)** | 20.24 (1.28) | 37.48 (1.28) | 11.32 (1.28) | 6.61 (1.28) | 0.0002 | 0.870 | 0.031 |
| **OXPHOS (CI)** | 47.50 (1.35) | 89.16 (1.35) | 13.39 (1.35) | 6.74 (1.35) | <0.0001 | 0.917 | 0.038 |
| **OXPHOS (CI+CII)** | 71.80 (1.28) | 148.87 (1.28) | 33.80 (1.28) | 27.69 (1.28) | 0.0001 | 0.154 | 0.039 |
| **ETS (CI+CII)** | 66.55 (1.27) | 141.03 (1.27) | 30.40 (1.27) | 25.63 (1.27) | <0.0001 | 0.103 | 0.034 |
| **ETS (CII)** | 32.96 (1.25) | 98.40 (1.25) | 22.01 (1.25) | 22.04 (1.25) | 0.003 | 0.0006 | 0.007 |
| **ROX** | 6.73 (1.23) | 21.87 (1.25) | 8.71 (1.25) | 4.52 (1.23) | 0.004 | 0.165 | 0.0002 |
| **FAT SUIT- O2 flux per mass corrected for citrate synthase abundance expressed in pmol/(s*mg)/CS expression** | | | | | | | |
| **Leak (PMOct)** | 73.19 (1.37) | 135.53 (1.37) | 80.09 (1.37) | 46.78 (1.37) | 0.185 | 0.854 | 0.038 |
| **OXPHOS (CI)** | 171.74 (1.40) | 322.37 (1.40) | 94.75 (1.40) | 47.69 (1.40) | 0.0020 | 0.913 | 0.037 |
| **OXPHOS (CI+CII)** | 259.62 (1.36) | 538.29 (1.36) | 239.18 (1.36) | 195.94 (1.36) | 0.0221 | 0.164 | 0.031 |
| **ETS (CI+CII)** | 240.64 (1.36) | 509.94 (1.36) | 215.08 (1.36) | 181.34 (1.36) | 0.0230 | 0.101 | 0.028 |
| **ETS (CII)** | 119.16 (1.33) | 355.78 (1.33) | 155.74 (1.33) | 155.90 (1.33) | 0.285 | 0.0008 | 0.006 |
| **ROX** | 24.35 (1.33) | 89.75 (1.33) | 61.68 (1.33) | 31.98 (1.33) | 0.860 | 0.158 | 0.0008 |

Data are presented as back-transformed LSmeans (SE).
