# Supplementary material for: The Preservation of Muscle Mitochondrial Machinery During Hypometabolic Hibernation in Scandinavian Brown Bears ( Ursus arctos )
Source: Acta Physiol (Oxf). 2026 Feb 23;242(4):e70177. doi: 10.1111/apha.70177 (PMC12926787; doi:10.1111/apha.70177)
Supplement: Supplementary file 7 — Table S5: Skeletal muscle isolated mitochondria respiratory rates measured in active and hibernating bears at 25°C. [file APHA-242-e70177-s005.docx]

**Supplemental Table 5. Skeletal muscle isolated mitochondria respiratory rates measured in active and hibernating bears at 25°C**

|  | **Summer physically active bears (n=8)** | **Winter hibernating**  **bears (n=8)** | **Season effect: p-value** |
| --- | --- | --- | --- |
| **CHO SUIT- O2 flux per mass uncorrected for mitochondrial DNA content expressed in pmol/(s*mg)** | | | |
| **Leak (PMG)** | 3.77 (1.16) | 1.25 (0.69) | 0.001 |
| **OXPHOS (CI)** | 14.02 (13.32) | 2.07 (2.41) | 0.001 |
| **OXPHOS (CI+CII)** | 16.12 (16.83) | 2.25 (2.01) | 0.059 |
| **ETS (CI+CII)** | 17.17 (16.61) | 2.39 (2.22) | 0.054 |
| **ETS (CII)** | 6.91 (4.63) | 3.06 (2.49) | 0.041 |
| **ROX** | 3.34 (0.81) | 1.97 (1.35) | 0.098 |
| **CHO SUIT- O2 flux per mass corrected for mitochondrial DNA content expressed in pmol/(s*mg)/mitochondrial protein content** | | | |
| **Leak (PMG)** | 1.92 (0.77) | 0.88 (0.48) | 0.026 |
| **OXPHOS (CI)** | 7.25 (7.49) | 1.41 (1.54) | 0.090 |
| **OXPHOS (CI+CII)** | 8.46 (9.58) | 1.47 (1.24) | 0.101 |
| **ETS (CI+CII)** | 8.97 (9.30) | 1.53 (1.31) | 0.053 |
| **ETS (CII)** | 3.66 (2.88) | 1.98 (1.52) | 0.034 |
| **ROX** | 1.84 (0.77) | 1.16 (1.01) | 0.317 |
| **FAT SUIT- O2 flux per mass uncorrected for mitochondrial protein content expressed in pmol/(s*mg)** | | | |
| **Leak (PMOct)** | 3.66 (2.17) | 1.32 (0.48) | 0.292 |
| **OXPHOS (CI)** | 6.73 (7.44) | 1.33 (0.58) | 0.232 |
| **OXPHOS (CI+CII)** | 15.39 (14.57) | 2.01 (1.66) | 0.003 |
| **ETS (CI+CII)** | 20.59 (21.52) | 2.53 (2.04) | 0.004 |
| **ETS (CII)** | 9.60 (4.22) | 3.93 (2.31) | 0.073 |
| **ROX** | 2.95 (2.05) | 2.22 (1.09) | 0.372 |
| **FAT SUIT- O2 flux per mass corrected for mitochondrial protein content expressed in pmol/(s*mg)/mitochondrial protein content** | | | |
| **Leak (PMOct)** | 2.01 (1.42) | 0.93 (0.37) | 0.031 |
| **OXPHOS (CI)** | 3.28 (3.70) | 0.93 (0.39) | 0.010 |
| **OXPHOS (CI+CII)** | 7.57 (6.98) | 1.39 (1.06) | 0.053 |
| **ETS (CI+CII)** | 9.94 (10.20) | 1.75 (1.35) | 0.070 |
| **ETS (CII)** | 5.45 (3.18) | 2.70 (1.53) | 0.018 |
| **ROX** | 1.86 (1.62) | 1.53 (0.68) | 0.324 |

Data are presented as back-transformed LSmeans (SE).
